# Widespread White Matter Abnormalities in Concussed Athletes Detected by 7T Diffusion Magnetic Resonance Imaging

**DOI:** 10.1089/neu.2023.0099

**Published:** 2024-07-17

**Authors:** Anna Gard, Evgenios N. Kornaropoulos, Maria Portonova Wernersson, Ia Rorsman, Kaj Blennow, Henrik Zetterberg, Yelverton Tegner, Alessandro De Maio, Karin Markenroth Bloch, Isabella Björkman-Burtscher, Hélène Pessah-Rasmussen, Markus Nilsson, Niklas Marklund

**Affiliations:** ^1^ Department of Clinical Sciences Lund, Neurosurgery, Neurology, Lund University, Lund, Sweden.; ^2^ Department of Clinical Sciences Lund, Diagnostic Radiology, Neurology, Lund University, Lund, Sweden.; ^3^ Department of Neurology, Rehabilitation Medicine, Memory Disorders and Geriatrics, Skåne University Hospital, Neurology, Lund University, Lund, Sweden.; ^4^ Department of Clinical Sciences Lund, Neurology, Lund University, Lund, Sweden.; ^5^ Department of Psychiatry and Neurochemistry, Institute of Neuroscience and Physiology, Sahlgrenska University Hospital, Mölndal, Sweden.; ^6^ Department of Neurodegenerative Disease, UCL Institute of Neurology, Queen Square, London, UK.; ^7^ UK Dementia Research Institute at UCL, London, UK.; ^8^ Hong Kong Center for Neurodegenerative Diseases, Clear Water Bay, Hong Kong, China.; ^9^ Department of Health, Education and Technology, Division of Health and Rehabilitation, Luleå University of Technology, Luleå, Sweden.; ^10^ Department of Radiological, Oncological and Pathological Sciences. Policlinico Umberto I, Sapienza University of Rome, Rome, Italy.; ^11^ Department of Clinical Sciences Lund, Lund University Bioimaging Center, Lund University, Lund, Sweden.; ^12^ Department of Radiology, Institute of Clinical Sciences, Sahlgrenska Academy, University of Gothenburg and Sahlgrenska University Hospital, Gothenburg, Sweden.; ^13^ Department of Clinical Sciences Lund, Neurosurgery, Lund University, and Skåne University Hospital, Lund, Sweden.

**Keywords:** biomarkers, neuropsychology, persistent post-concussion symptoms, 7T MRI, sports-related concussion

## Abstract

Sports-related concussions may cause white matter injuries and persistent post-concussive symptoms (PPCS). We hypothesized that athletes with PPCS would have neurocognitive impairments and white matter abnormalities that could be revealed by advanced neuroimaging using ultra-high field strength diffusion tensor (DTI) and diffusion kurtosis (DKI) imaging metrics and cerebrospinal fluid (CSF) biomarkers. A cohort of athletes with PPCS severity limiting the ability to work/study and participate in sport school and/or social activities for ≥6 months completed 7T magnetic resonance imaging (MRI) (morphological T1-weighed volumetry, DTI and DKI), extensive neuropsychological testing, symptom rating, and CSF biomarker sampling. Twenty-two athletes with PPCS and 22 controls were included. Concussed athletes performed below norms and significantly lower than controls on all but one of the psychometric neuropsychology tests. Supratentorial white and gray matter, as well as hippocampal volumes did not differ between concussed athletes and controls. However, of the 72 examined white matter tracts, 16% of DTI and 35% of DKI metrics (in total 28%) were significantly different between concussed athletes and controls. DKI fractional anisotropy and axial kurtosis were increased, and DKI radial diffusivity and radial kurtosis decreased in concussed athletes when compared with controls. CSF neurofilament light (NfL; an axonal injury marker), although not glial fibrillary acidic protein, correlated with several diffusion metrics. In this first 7T DTI and DKI study investigating PPCS, widespread microstructural alterations were observed in the white matter, correlating with CSF markers of axonal injury. More white matter changes were observed using DKI than using DTI. These white matter alterations may indicate persistent pathophysiological processes following concussion in sport.

## Introduction

Acute symptoms following sports-related concussion (SRC) include physical, cognitive, and emotional disturbances that commonly subside within 7–14 days after injury.^1^ However, 10–30% of athletes develop persistent post-concussive symptoms (PPCS),^2,3^ which include neurocognitive deficits and impaired long-term mental health and quality of life.^4,5^ Although there is no consensus or gold standard for the definition of PPCS, which varies among studies, the diagnosis is often based on symptom rating (e.g. using self-report rating scales) with symptoms persisting beyond the normal recovery time.^2^

There are growing concerns that several mild traumatic brain injuries (mTBI) and SRCs may initiate a long-term neurodegenerative process in the brain, associated with white matter (WM) pathology.^6,7^ However, often there is an absence of gross structural pathology on conventional neuroimaging.^8^ Presumably, subtle abnormalities may be detected by advanced magnetic resonance imaging (MRI) techniques at higher field strengths. Diffusion tensor imaging (DTI) is an imaging technique that sensitizes the images to the microstructure of brain tissue by probing the diffusional motion of water molecules.^9^ Diffusion kurtosis imaging (DKI) is an extension to DTI that may be more sensitive to microstructural changes. It not only provides measures of the average diffusivity and anisotropy, but also measures the diffusional kurtosis, which reflects the degree to which the microstructure is heterogeneous.^10,11^ DTI and DKI have been evaluated in SRC^12–15^ and PPCS^16,17^ and have been found to provide imaging metrics related to prolonged symptoms^18^ and impaired results on neuropsychology tests.^12,19–23^ In fact, the risk of developing PPCS was predicted by DTI and DKI metrics at 72 h post-injury,^18^ and DTI and DKI may provide neurobiological and microstructural correlates to SRC-induced symptoms. However, none of the previous DTI or DKI studies examined the use of ultra-high field strength (≥ 7T). At 7T, the signal-to-noise ratio (SNR) and contrast-to-noise ratio (CNR) of DTI are supposed to be enhanced when compared to 3T-DTI.^24–26^

Apart from imaging, there is also a vast interest in biomarkers for SRC, aimed at diagnosis, assessment of injury severity, and outcome prediction. Increased levels of neurofilament light (NfL), a component of the cytoskeleton of large-caliber myelinated axons, correlate with persistent symptoms following SRC.^27^ Glial fibrillary acidic protein (GFAP), a biomarker of astrogliosis,^28^ also increases over the short and long term following SRC.^29,30^ Acute blood biomarker levels have been shown to correlate with WM atrophy and DTI measures of axonal injury,^30^ and biomarker levels are associated with DTI metrics.^31^ However, the association between cerebrospinal fluid (CSF) GFAP or NfL levels and DKI parameters has not previously been evaluated.

The multifaceted symptoms experienced by PPCS athletes, caused by heterogeneous injury mechanism, cannot be explained by a single injury mechanisms and multimodal assessment including neuropsychology. CSF biomarker and neuroimaging studies are required. Therefore, in the present explorative study, we evaluated the neurocognitive and mental health outcomes in athletes with SRC and PPCS for >6 months, and explored measures of atrophy using 7T MRI volumetry and measures of tissue microstructure using 7T DTI and DKI. For inclusion, symptom severity should prevent participation in sports, and limit work, school, or regular social activities. We choose to include DTI and DKI metrics in all WM areas of the brain to provide a study of WM microstructure in SRC athletes to assess the additive value of ultra-high field DTI and DKI on mild TBI research. We also evaluated CSF biomarkers of astrogliosis and axonal injury.

## Methods

### Ethics

The study was approved by the Regional Ethics Committee of Lund University, Lund, Sweden (Dnr 2017/1049) and conducted in accordance with the Declaration of Helsinki. All participants signed a written consent form.

### Study population

We included adult elite athletes previously participating competitively at a high national level with a history of one or more SRCs and with ongoing PPCS ≥6 months. The symptom duration was selected based on the time that may be required for phosphorylated tau following traumatic brain injuries,^32^ and on our previous work using tau-positron emission tomography (PET).^33^ The PPCS athletes were included if they experienced debilitating symptoms with a severity that prevented participation in sports, and limited work, school, or regular social activities. A specific number of symptoms was not a criterion, instead the inclusion criteria were based on the severity of symptoms and the impact of them on the social, academic, and professional life of the athlete. Healthy non-competing controls, exercising three or more times per week for the last 5 years or longer were matched with the SRC athletes concerning sex and age, and were included as controls. Controls and PPCS athletes were specifically interviewed regarding any history of previously diagnosed or suspected SRC after explaining the definition of concussion, or other traumatic brain injuries requiring hospitalization. Had such brain injuries occurred in the past, the controls would be excluded from the study. The controls were all participating full time in higher education or work. Exclusion criteria were: age <18 years, unable to complete an MRI, and prior or current neurological or psychiatric disorders. The subjects were recruited by advertising and via rehabilitation medicine, sports team physicians, physiotherapists, word of mouth, personal contacts, and contacts within Swedish sports societies. One of the researchers (A.G.) interviewed and informed potential study participants. The SRC athletes completed, prior to but on the day of the MRI investigation, the Sport Concussion Assessment Tool 5th edition (SCAT5)^34^ which evaluates 22 post-concussion symptoms and has a maximum severity score of 132.

### Neuropsychological assessment

Neuropsychological assessment included a semi-structured interview, psychometric testing, and self-report questionnaires that were selected based on the cognitive and emotional symptoms commonly associated with PPCS.^4^ SRC athletes and controls were compared on all measures. In addition, cognitive results were assessed in terms of deviation from age-related published norms. The following measures were included: Repeatable Battery for the Assessment of Neuropsychological Status (RBANS), d2 test of attention, Symbol Digit Modalities Test (SDMT), Digit Span – Wechsler Adult Intelligence Scale (WAIS)-IV, Hospital Anxiety Depression Scale (HADS), Mental Fatigue Scale (MFS), Life Satisfaction Questionnaire (LiSat)11, and Behaviour Rating Inventory of Executive Function-Adult version (BRIEF-A). Descriptions of these measures and their sensitivity to difficulties associated with PPCS or TBI are as follows.

### RBANS

RBANS is a psychometric test battery consisting of 12 subtests organized into five index scores and a total global score.^35^ It measures several cognitive domains of interest in mTBI: immediate memory, attention, language, visuospatial/constructional ability, and delayed memory. RBANS was designed for the evaluation of cognitive deficits in neurodegenerative diseases^36^ and has been validated for TBI patients.^37^

### d2

The d2 test of attention (d2) test is a timed psychometric measure of selective and sustained attention and processing speed.^38^ Subjects are asked to overstrike the letter d with a specified arrangement of dots, on a sheet containing 14 rows with 47 ps and ds with different arrangements of dots. The test has been used to identify residual cognitive deficits in young adults with a history of concussions.^39^

### SDMT

SDMT is a psychometric test that taps into many cognitive functions including working memory, attention, and, especially, information processing speed.^40^ In the test, subjects are asked to translate as many as possible geometric symbols into numbers, during a restricted amount of time. SDMT is a widely used, reliable, and valid measure for detecting cognitive impairment and decline in a number of neurological disorders and brain injuries, including TBI.^41^

### Digit span - WAIS-IV

Digit span from the WAIS-IV is a subtest composed of three tasks: Forward Digit span, Backward Digit span, and Sequencing.^42^ In the Forward Digit span task, participants are read a sequence of numbers and asked to repeat these in order whereas in the Backward Digit span task the numbers are to be repeated in reverse order. In the Sequencing task, the random numbers are to be repeated in ascending order. The Digit Span subtest measures auditory attention, short-term retention, and working memory, and has been shown to differentiate patients with TBI from matched controls.^43^

### HADS

The HADS is a self-reported measure of anxiety (HADS-A) and depression (HADS-D). Scores are graded in four rankings on a Likert scale. High scores imply a high level of self-perceived symptoms. Scores of eight or more, for HADS-A and HADS-D, respectively, indicate clinically significant levels of anxiety and depression.^44^ These tests have been used in detecting these symptoms in PPCS.^45^

### MFS

MFS^46^ is a self-report measure of typical and associated symptoms of mental fatigue following acquired brain injuries, including mTBI. Typical symptoms of mental fatigue include acute loss of energy, difficulties with maintaining concentration, prolonged recovery time, and time-dependent access of mental energy. Associated symptoms include mood swings, irritability, sensitivity to stress, impaired memory, sleep disturbances, and sensitivity to noise and/or light. Symptoms are rated on intensity, frequency, and duration, and graded in seven rankings on a Likert scale. High scores indicate a high level of self-perceived mental fatigue. A cutoff of 10.5 points is indicative of mild mental fatigue and predicts a decline in cognitive processing speed in PPCS.^47^

### LiSat-11

The 11-item LiSat (LiSat-11) contains a self-report assessment of global satisfaction (“Life as a whole”) and four domain-specific satisfaction levels of social relations, health, leisure time, and work/economy.^48^ Every question has six alternatives, ranked on an ordinal scale, with high rating indicates high level of satisfaction. Life satisfaction is an individual's contentment with life, and usually reflects a feeling of achievement regarding expected goals, ambitions, and performance. LiSat-11 has been used in previous research in TBI^49^ to identify long-term consequences on life satisfaction.

### BRIEF-A

The BRIEF-A^50^ is a standardized self-report measure that captures the participant's views of their everyday executive functioning and self-regulation. The form has 75 questions with nine subscales: Inhibit, Self-monitor, Plan/organize, Shift, Initiate, Task monitor, Emotional control, Working memory, and Organization of materials. The scales form two indexes: Behaviour Regulation Index (BRI) and Metacognition Index (MI), as well as the summarizing Global Executive Composite (GEC). BRIEF-A also includes three scales of self-report validations, which estimate the ability to answer the form correctly. Scores are graded in three rankings on a Likert scale, with high scores indicating worse executive function. BRIEF-A has previously been shown to indicate the extent of executive dysfunction in retired contact sport athletes.^51^

### 7T MRI

Imaging was performed on a 7T Philips Achieva system. The MRI protocol comprised three sequences: a 3D T1-weighted magnetization-prepared rapid gradient-echo (MPRAGE) sequence (field of view [FOV]: 230 × 230 × 180 mm^3^, resolution 0.80 × 0.80 × 0.80 mm^3^, repetition time [TR]/echo time [TE]: 8.00/1.97 ms); a diffusion-weighed imaging (DWI) sequence for DTI (FOV: 224 × 224 × 110 mm^3^, resolution 2 × 2 × 2 mm^3^, and TR/TE: 9200/65 ms) in which the diffusion encoding was applied in 6 directions with b = 100 sec/mm^2^ and 30 directions with b = 1000 s/mm^2^ and; a DWI sequence for DKI (FOV: 224 × 224 × 120 mm^3^, resolution 2 × 2 × 2 mm^3^, and TR/TE: 9800/76 ms) in which the diffusion encoding in 6 directions with b = 100 sec/mm^2^, 6 with b = 500 sec/mm^2^, 10 with b = 1000 sec/mm^2^, and 30 with b = 2000 sec/mm^2^. For both protocols, two b = 0 sec/mm^2^ volumes were acquired with opposing polarities of the phase-encode blips for use in distortion correction.

### Volumetrics

**FIG. 1.**
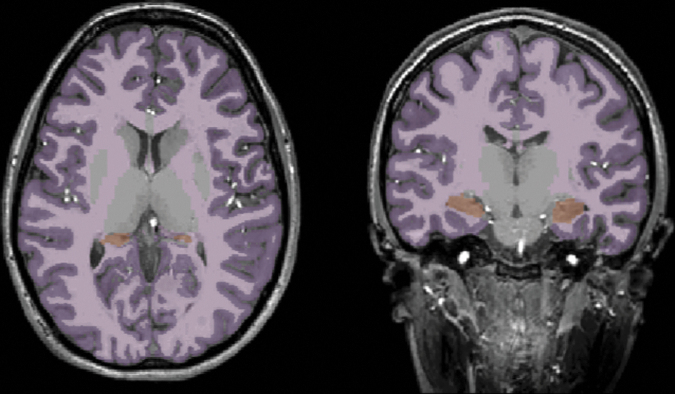
Brain volumetry. Depiction of the performance of FreeSurfer segmentation on a T1-weighed image. Supratentorial gray matter (purple), white matter (pink), and hippocampi (orange) are portrayed in an axial (left) and coronal (right) view.

For volumetry, regions of interest were segmented from the T1-weighed images using FreeSurfer^52^ (version 7.1.1 found at http://surfer.nmr.mgh.harvard.edu). Volumes of the supratentorial WM and gray matter (GM), and hippocampi were analyzed (Fig. 1). An expert reader (E.N.K.) inspected each three-dimensional segmentation image for points of misclassification of WM, GM, and pial surface boundaries in three separate editing steps.^53^ Following initial labeling, images were inspected for WM omissions and control points were added, extending the boundaries of WM segmentation to accurately incorporate WM. Subsequently, the images were inspected for any meningeal residuals within the pial surface which, if present, was manually removed. Finally, an inspection of potential erroneous classification of WM as GM or vice versa was performed, and edits were made to the parcellation image to redraw WM and GM boundaries. In the case of B1+ inhomogeneity severely affecting the image, causing a drop in SNR, the specific subject was excluded from the morphology analysis, as none of the abovementioned steps would mitigate the issue. All images were verified by two trained supervisors (M.N. and N.M.) to ensure that edits were implemented correctly.

### DTI and DKI

**FIG. 2.**
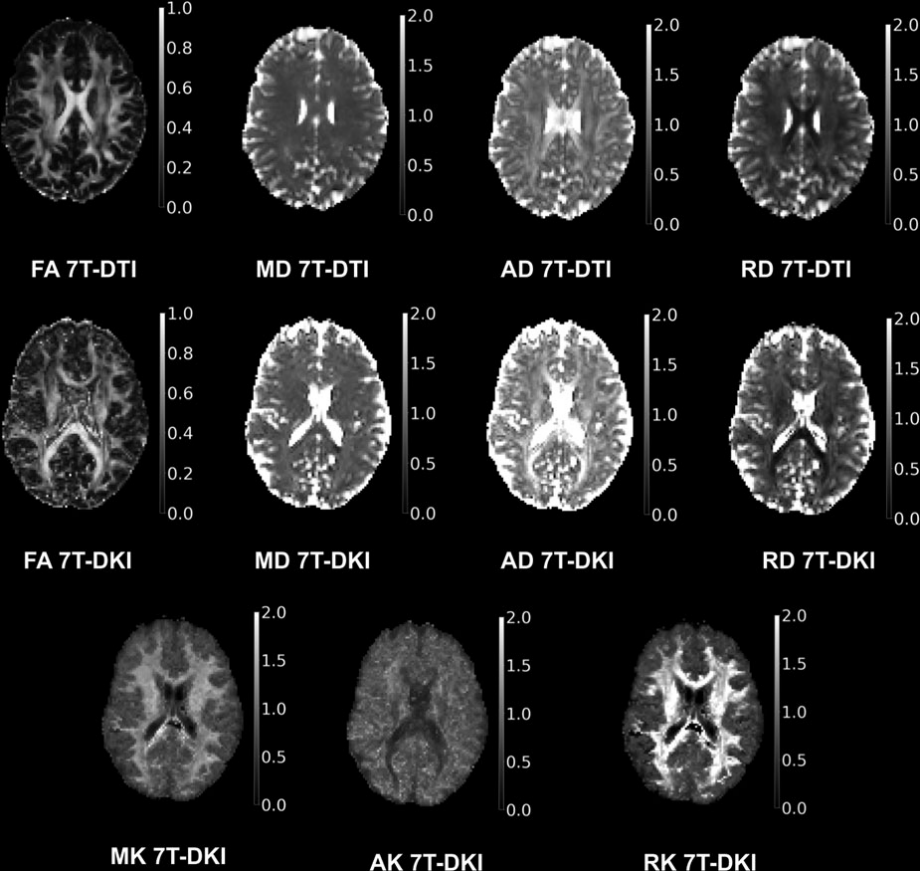
7T diffusion tensor imaging (DTI) and diffusion kurtosis imaging (DKI) maps. Axial maps for the metrics of DTI and DKI. Included metrics are fractional anisotropy (FA), mean diffusivity (MD), axial diffusivity (AD), and radial diffusivity (RD) for DTI, and FA, MD, AD, RD, mean kurtosis (MK), axial kurtosis (AK), and radial kurtosis (RK) for DKI.

DTI and DKI processing comprised four steps: de-noising, correction for Gibbs-ringing artefacts, brain extraction, and correction of distortions caused by head motion and eddy currents. De-noising was performed using Marchenko–Pastur principal component analysis.^54^ For mitigating Gibbs-ringing artefacts we used the method proposed by Kellner and coworkers.^55^ Brain extraction was performed with the use of a T1-weighted brain mask that was extracted via the advanced normalization tools (ANTs) brain extraction scheme.^56^ Once a brain mask was extracted from the T1-weighted volume, the b-zero volume (b = 0 sec/mm^2^ ) was rigidly registered onto the T1-weighted volume using the ANTs rigid registration scheme,^57^ and then the inverse transformation matrix was used to bring the brain mask onto the DTI or DKI space. Motion and eddy currents correction was applied using the eddy method provided by FSL.^58^ Geometric distortions were corrected using topup by FSL.^59^ In cases of remaining artefacts not mitigated by the aforementioned processing steps, the specific scan was excluded from the analysis. DTI parameters were estimated through DTIFIT in FSL using weighted linear least squares.^60^ DKI parameters were computed using the package dipy and its module DiffusionKurtosisModel, using weighted linear least squares in the fitting.^10^ For both DTI and DKI, the two b-zero volumes were not included in the model fitting. Median filtering with a kernel size of three was applied to the three kurtosis parameters (i.e., mean, axial, and radial kurtosis), following their initial computation, in order to mitigate the issue with the black voxels in DKI.^61^ To obtain tract-specific diffusion parameter values, WM tract segmentation was performed using TractSeg,^62^ which automatically segments 72 major WM tracts. All 72 tracts were analyzed in this study. The fractional anisotropy (FA), mean diffusivity (MD), axial diffusivity (AD), and radial diffusivity (RD) were analyzed for DTI and FA, MD, AD, RD, mean kurtosis (MK), axial kurtosis (AK), and radial kurtosis (RK) were analyzed for DKI (Fig. 2). For all metrics, the tracts were summarized and divided by 72, to generate a mean value for all 11 metrics called “single WM region of interest (ROI).”

### CSF biomarkers

In SRC athletes, 5 mL CSF was drawn by a routine lumbar puncture. The CSF was centrifuged at 3000 rpm at 4°C for 10 min and stored at −80°C. The samples were sent to the Clinical Neurochemistry Laboratory, University of Gothenburg, Sweden on dried ice.

CSF NfL, and GFAP concentrations were measured using an in-house enzyme-linked immunosorbent assay (ELISA).^63,64^ All analyses were performed by technicians blinded to clinical data in one round of experiments. Intra-assay coefficients were <10%. Reference values were provided by the Gothenburg Laboratory.

### Statistical analysis

Data were statistically analyzed in the Statistical Package for the Social Sciences (SPSS Inc., Version 25, IBM, New York). Data were analyzed for normal or skewed distribution using the Shapiro–Wilk test and histograms and presented using means and standard deviations (SD) if normally distributed and with medians and interquartile ranges (IQR) if skewed, nominal, or categorical. Because of the limited sample size, non-parametric tests were chosen for all analyses; χ^2^ tests if categorical or binominal, Mann–Whitney *U* test if the data were nominal or continuous, and for correlations, Spearman's coefficient (r_s_) were calculated.

For calculating differences in subject characteristics, if normally distributed and with medians and IQRs if skewed, nominal, or χ^2^ tests were used for sex, occupation status, and housing situation, and Mann–Whitney *U* tests were used for age and years of education. Neuropsychological measures were analyzed with Mann–Whitney *U* tests, using raw scores for comparisons between SRC athletes and controls. In addition, to determine any deviations from normal populations, raw scores from the cognitive tests (RBANS, d2, SDMT, and WAIS-IV) and the self-report inventory of executive functioning, BRIEF-A, were converted into standard Z-scores, derived from test-specific age-related published norms. Manifestation of cognitive difficulties/deficits was defined by results ≤ −1.5 Z; that is, 1.5 SD below normative means for each measure,^65^ corresponding approximately to <6.7% in the general population. Volumetric and diffusion data were compared across groups using Mann–Whitney *U* tests. The significance threshold was set to 0.05.

Corrections for multiple comparison were not done in this explorative study. To mitigate the problem of multiple comparisons, however, we compared the number of significant tests to the corresponding one computed under the null hypothesis using a permutation test, as previously proposed.^66,67^ In this analysis, the test statistic was defined as the number of tracts that displayed significant differences in their medians. For each permutation, the labels of the two groups were randomly permuted after which a *U* test was performed for each tract. Using 10^4^ permutations, this generated a distribution of the number of tracts with significant differences, under the null hypothesis. The exact *p* value was computed as the relative fraction for which the number of significant tracts from the permutation analysis was higher than the number of significant tracts with the true labels. Using the results of the permutation analysis, we also computed the 95th percentile of the number of tracts that was significant under the null hypothesis. This number can be considered as a significance threshold: for parameters with fewer significant tracts, all significant tracts for that parameter may be considered as false positives. For parameters with a higher number of significant tracts, some but not all of the significant tracts can be considered as false positives. The permutation analysis was repeated for significance thresholds (ɑ) in the per-tract *U* tests of 0.05 and 0.01.

Effect sizes were calculated for differences between SRC athletes and controls in the DTI and DKI metrics. Effects sizes above 0.64 or below −0.64 were considered significant, as the 95% confidence interval of effect sizes in the absence of a true effect spans this range for our group sizes.^68^

## Results

### Study population

Forty-four subjects were included, 22 athletes with previous SRCs, and 22 healthy age- and sex-matched controls (Table 1). One SRC athlete did not complete the MRI and five controls did not complete the neuropsychology testing and self-reported measures. These subjects were excluded from the specific analyses. One athlete and one control were on antidepressants at time of the investigations. There were no smokers among the study participants.

**Table 1.** Subject Characteristics

	SRC athletes,* n* = 22	Controls,* n* = 22
Male sex, % (*n*), *p* = 0.353	68% (15)	55%, (12)
Age, mean (SD), *p* = 0.580	27 (6.4) years	26 (5.0) years
**Education, mean (SD), *p<*0.001**	**13 (1.6) years**	**16 (2.6) years**
Works full time, % (*n*), *p* = 0.340	27% (6)	41% (9)
Works part time, % (*n*), *p* = 0.472	27% (6)	18% (4)
Studies, % (*n*), *p* = 0.112	23% (5)	45% (10)
**On sick leave, % (*n*), *p*<0.001**	**50% (11)**	**0% (0)**
Lives alone, % (*n*), *p = 0.353*	45% (10)	32% (7)
Sports (*n*)	Ice hockey (8), soccer (4), karate (4), handball (2), indoor hockey (2), wrestling (1), and endurance riding (1)	Running, weight lifting, cycling, and swimming
Years of sports practice, mean (SD)	18 (5.1)	-
Number of SRCs, mean (range)	5 (1-20)	-
Age at first SRC, mean (SD)	18 (5.2) years	-
Time from first SRC, mean (SD)	8.9 (6.8) years	-
Time from last SRC, mean (SD)	2.6 (3.2) years	-
Rehab, yes % (*n*)	14% (3)	-
SCAT5 symptom severity score, median (IQR)	64 (44.5-81.5)	-
SCAT5 number of symptoms, median (IQR)	20 (20-22)	-

Demographic details on the 44 participants of the present study. The subjects' employment rates, studies, and sick leave times vary from 0 to 100%, with a possible combination of these. The sports that the sports-related concussion (SRC) athletes were involved in are listed in declining order. As controls were commonly involved in multiple athletic activities, specific numbers are not listed. The Sport Concussion Assessment Tool 5th edition (SCAT5) has a maximum symptom severity score of 132 and a maximum number of symptoms of 22. Significantly different *p* values at ≤ 0.05 are shown in bold.

SD, standard deviation and IQR, interquartile range.

The mean age was 26 (range 18–43) years and 61% were males. The groups were similar in most of the demographics, although the SRC athletes had less education (13 vs. 16 years; *p* < 0.001) and were more frequently on sick leave (50 vs. 0%; *p* < 0.001). The SRC athletes reported many and severe symptoms on the SCAT5, with a median of 20 symptoms (max 22) and a symptom severity score of 64 (max 132). Subject characteristics and SRC details are listed in Table 1.

### Neuropsychological assessment

#### Psychometric tests

**FIG. 3.**
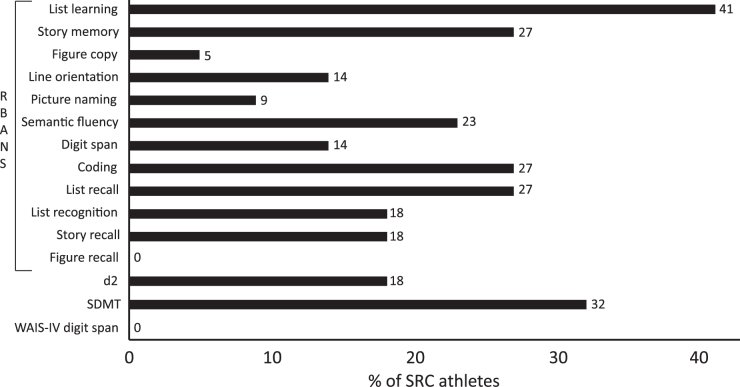
Psychometric neuropsychology tests. Percent of sports-related concussion (SRC) athletes with scores Z≤-1.5 on the psychometric neuropsychology tests; Repeatable Battery for the Assessment of Neuropsychological Status (RBANS), the d2 test of attention (d2), Symbol Digit Modalities Test (SDMT), and Wechsler Adult Intelligence Scale – fourth edition (WAIS-IV) digit span. After each column the percent is stated.

SRC athletes performed worse than averaged norms on all but four psychometric tests (Table 2) and 12 out of 22 of the SRC athletes showed deficits in at least one cognitive domain i.e. Z≤ −1.5 (Fig. 3). On the RBANS Global Score, 41% (9/22) of SRC athletes scored Z≤ −1.5, and RBANS list learning was the most sensitive test with 41% (9/22) having a Z-score below cutoff. When comparing SRC athletes with the controls from our study, SRC athletes performed significantly worse on all but one of the psychometric measures (Table 2)

**Table 2.** Neuropsychology Performances

	SRC athletes,* n* = 22		Controls,* n* = 17		*n *(%) of SRC athletes deviating from norms	*p *value SRC athletes vs. controls
	Z-score, median (IQR)	Raw score, median (IQR)	Z-score, median (IQR)	Raw score, median (IQR)		
Psychometric tests						
RBANS						
Immediate memory	-1.24 (-2.08 – -0.17)	79.0 (65.0 – 97.0)	0.13 (-0.92 – 0.84)	102 (84.5 – 114)	8 (36)	
List learning	-1.23 (-2.45 – 0.05)	27.5 (23.0 – 32.5)	0.18 (-0.46 – 0.44)	33.0 (30.5 – 34.0)	9 (41	
Story memory	-0.81 (-1.74 – 0.11)	17.5 (15.0 – 20.0)	-0.26 (-0.63 – 0.85)	19.0 (18.0 – 22.0)	6 (27)	
Visuospatial functions	-0.29 (-1.17 – 0.29)	96.0 (83.0 – 105)	0.12 (-0.29 – 0.46)	102 (96.0 – 107)	3 (14)	
Figure copy	0.41 (-0.42 – 0.42)	20.0 (19.0 – 20.0)	0.42 (-0.02 – 0.42)	20.0 (19.5 – 20.0)	1 (5)	
Line orientation	-0.06 (-1.03 – 0.78)	18.5 (17.0 – 20.0)	0.39 (-0.17 – 0.94)	19 .0 (18.0 – 20.0)	3 (14)	
Verbal functions	-1.10 (-1.58 – -0.34)	83.5 (68.5 – 98.0)	0.32 (-0.34 – 1.01)	105 (95.0 – 116)	5 (23)	
Picture naming	0.40 (0.40 – 0.40)	10.0 (10.0 – 10.0)	0.40 (0.40 – 0.40)	10.0 (10.0 – 10.0)	2 (9)	
Semantic fluency	-1.11 (-1.49 – -0.45)	16.5 (14.5 – 20.0)	0.30 (-0.45 – 0.77)	24.0 (20.0 – 26.5)	5 (23)	
Attention functions	-1.06 (-2.03 – -0.12)	83.5 (68.5 – 98.0)	-0.06 (-0.64 – 0.52)	99.0 (90.0 – 108)	8 (36)	
Digit span	-0.41 (-1.09 – 0.50)	9.50 (8.00 – 11.5)	-0.18 (-0.86 – 0.73)	10.0 (8.50 – 12.0)	3 (14)	
Coding	-0.78 (-1.84 – -0.19)	46.0 (37.5 – 53.0)	-0.09 (-0.40 – 0.45)	54.0 (51.0 – 59.0)	6 (27)	
Delayed memory	-0.50 (-1.02 – -0.25)	92.0 (84.0 – 96.0)	0.26 (-0.22 – 1.15)	104 (96.5 – 118)	4 (18)	
List recall	-0.55 (-1.97 – -0.06)	7 (4.75 – 8.00)	0.53 (-0.65 – 0.82)	9.00 (7.00 – 9.50)	6 (27)	
List recognition	0.36 (-0.88 – 0.38)	20 (19.0 – 20.0)	0.38 (0.38 – 0.38)	20.0 (20.0 – 20.0)	4 (18)	
Story recall	0.19 (-1.38 – 0.50)	10.5 (8.00 – 11.0)	0.50 (-0.13 – 0.13)	11.0 (10.0 – 12.0)	4 (18)	
Figure recall	0.00 (-1.07 – 0.36)	17 (14.0 – 18.0)	0.36 (0.00 – 0.71)	18.0 (17.0 – 19.0)	0 (0)	
Global score	-1.29 (-2.28 – -0.20)	79.5 (64.5 – 96.0)	1.19 (-0.17 – 0.85)	102 (96.5 – 112)	9 (41)	<0.001
d2	-0.25 (-0.60 – 0.70)	369 (338 – 436)	1.25 (0.65 – 2.10)	475 (432 – 535)	4 (18)	<0.001
SDMT	-0.79 (-1.71 – -0.29)	47 (42 – 54)	0.20 (-0.14 – 0.44)	57.5 (54.0 – 61.0)	7 (32)	<0.001
WAIS-IV digit span	-0.39 -0.75 – 0.50)	24 (22 – 27.5)	0.32 (-0.39 – 0.59)	25.0 (22.5 – 29.0)	0 (0)	0.639
Self-report measures					**-from cut-off**	
HADS-A	-	9.0 (6.0-12.0)	-	3.0 (2.0 – 4.0)	13 (59)	<0.001
HADS-D	-	6.0 (4.75 – 8.50)	-	1.0 (0.5 – 1.0)	5 (23)	<0.001
MFS	-	21.0 (17.0 – 25.0)	-	4.5 (1.25-5.75)	21 (95)	<0.001
LiSat-11	-	42.0 (38.0 – 48.5)	-	56.0 (51.0 – 61.0)	18 (82)	<0.001
BRIEF-A					**-from norms**	
Metacognition index	1.22 (0.60 – 1.59)	74.0 (68.5 – 82.0)	-0.52 (-0.86 – -0.05)	54.0 (49.5 – 59.5)	6 (27)	
Behavior regulation index	1.20 (0.72 – 1.61)	56.5 (50.5 – 60.5)	-0.59 (-1.06 – -0.08)	37.0 (32.5 – 42.5)	6 (27)	
Global executive composite	0.98 (0.65 – 1.77)	129 (120 – 141)	-0.61 (-0.93 – -0.30)	89.0 (84.5 – 100)	9 (41)	<0.001

Psychometric tests include results converted into Z-scores from age-related norms and raw scores. Number and percent of sports-related concussion (SRC) athletes deviating from norms refers to ±1.5 Z, and from test-specific cutoffs; HADS-A > 8, HADS-D >8, MFS >10.5, and LiSat-11 “not satisfied with life as a whole.” The presented *p* values represent differences in raw total scale-score between SRC athletes and controls.

IQR, inter-quartile range. RBANS, Repeatable Battery for the Assessment of Neuropsychological Status (12 subtests organized into five index scores and a global score); d2, d2 test of attention; SDMT. Symbol Digit Modalities Test; Digit span - WAIS-IV, Wechsler Adult Intelligence Scale – fourth edition. Self-report measures are presented as raw scores and include HADS-A, The Hospital Anxiety and Depression Scale – anxiety; HADS-D, the Hospital Anxiety and Depression Scale – Depression; MFS, the Mental Fatigue Scale; LiSat-11, the 11-item Life Satisfaction questionnaire; BRIEF-A, the Behaviour Rating Inventory of Executive Function-Adult version with two index scores and a global score – includes raw scores and Z-scores.

#### Self-reported measures

Self-rated symptoms of anxiety (HADS-A), depression (HADS-D), and mental fatigue (MFS) significantly exceeded those reported by matched controls (*p* < 0.001; Table 2). A majority of SRC athletes, 59%, scored above levels indicative of clinical anxiety on HADS-A,^44^ and all SRC athletes reported symptoms of mental fatigue above normal.^47^ On the LiSat-11, 18 (82%) of the SRC athletes reported not being satisfied with life (defined by the four lowest grades on assessment of global satisfaction – “life as a whole”),^49^ with the least satisfaction being in the psychological health domain (0 %). On self-reported executive function (BRIEF-A), the SRC athletes rated significantly more problems than controls (*p* < 0.001) and deviated significantly (≤ −1.5 Z) from normative reference data.^50^

### 7T MRI

Images were assessed by a neuroradiologist (I.B.-B.), and no gross abnormalities were detected. The SRC athletes had all previously undergone routine neuroimaging (computed tomography or 1.5/3T MRI) following their SRC/SRCs, and these images were reported to the research team as normal. Volumetric segmentation was successfully performed in 19 athletes (missing data *n* = 1, artefacts *n* = 2) and 19 controls (artefacts *n* = 3). DTI evaluation was successfully performed in 20 athletes (missing data *n* = 2) and 21 controls (excessive head motion *n* = 1) and DKI evaluation was performed in 19 athletes (missing data *n* = 3) and 20 controls (artefacts *n* = 1, excessive head motion *n* = 1).

#### Volumetrics

There were no significant differences in WM volume, GM volume, or hippocampal volume between SRC athletes and controls. Supratentorial WM volume was 476 mL (IQR 445–552) in SRC athletes and 487 mL (IQR 464–560) in controls (*p* = 0.589); GM volume was 555 mL (IQR 555–611) in athletes and 598 mL (IQR 538–616) in controls (*p* = 0.737); hippocampal volume was 8.07 mL (IQR 7.69–8.75) in athletes and 8.08 mL (IQR 7.43–8.26) in controls (*p* = 0.672).

#### DTI and DKI- single WM ROI

The single WM ROI values differed significantly in four metrics; DKI FA where SRC athletes had higher values than controls (median 0.459 [IQR 0.448–0.464] vs. 0.438 [0.436–0.449], *p* < 0.001), DKI RD where SRC athletes had lower values than controls (0.698 [0.681–0.711] vs. 0.710 [0.700–0.728], *p =* 0.011), DKI AK where SRC athletes had higher values than controls (0.861 [0.851–0.872] vs. 0.842 [0.827–0.860], *p =* 0.023), and DKI RK where SRC athletes had lower values than controls (1.121 [1.008–1.182] vs. 1.185 [1.134–1.263], *p = 0.026*; Table 3). No significant differences between controls and SRC athletes were found for any single WM ROI in the DTI metrics.

**Table 3.** Global WM Metric Values

		SRC athletes, median (IQR)	Controls, median (IQR)	*p *value
DTI	FA	0.414 (0.403-0.421)	0.412 (0.397-0.420)	0.322
	MD	0.778 (0.755-0.794)	0.779 (0.769-0.795)	0.335
	AD	1.135 (1.122-1.151)	1.140 (1.128-1.160)	0.389
	RD	0.599 (0.572-0.610)	0.600 (0.585-0.619)	0.262
DKI	FA	0.459 (0.448-0.464)	0.438 (0.436-0.449)	**<0.001**
	MD	0.940 (0.918-0.957)	0.948 (0.935-0.959)	0.144
	AD	1.436 (1.394-1.448)	1.413 (1.401-1.447)	0.822
	RD	0.698 (0.681-0.711)	0.710 (0.700-0.728)	**0.011**
	MK	0.949 (0.898-0.976)	0.966 (0.945-1.017)	0.053
	AK	0.861 (0.851-0.872)	0.842 (0.827-0.860)	**0.023**
	RK	1.121 (1.008-1.182)	1.185 (1.134-1.263)	**0.026**

The diffusion values in medians and inter quartile ranges (IQR) for the sports-related concussion (SRC) athletes (diffusion tensor imaging [DTI] *n* = 20 and diffusion kurtosis imaging [DKI] *n* = 19) and controls (DTI *n* = 21 and DKI *n* = 20). The global white matter (WM) metric values and the *p* values of the group comparisons are presented for the investigated metrics. DTI metrics are fractional anisotropy (FA), mean diffusivity (MD), axial diffusivity (AD) and radial diffusivity (RD), and DKI metrics are FA, MD, AD, RD, mean kurtosis (MK), axial kurtosis (AK) and radial kurtosis (RK). Significant *p* values ≤0.05 are bolded.

#### DTI and DKI- tract-specific analysis

There was a significant difference between SRC athletes and controls in 16% of the 72 examined tracts analyzed by DTI metrics and in 35% of the tracts analyzed by DKI. In total, 28% of all comparisons showed significant differences between SRC athletes and controls (i.e., 224 out of 792 comparisons in total, as one test was performed for each of the 72 tracts and each of the 11 metrics). DTI FA differed in three tracts, DTI MD differed in 18 tracts, DTI AD differed in 14 tracts, DTI RD differed in 12 tracts, DKI FA differed in 52 tracts, DKI MD differed in 19 tracts, DKI AD differed in zero tracts, DKI RD differed in 36 tracts, DKI MK differed in six tracts, DKI AK differed in 35 tracts, and DKI RK differed in 29 tracts (Table S2). For details in DTI and DKI metrics for the specific tracts, see Supplementary Tables S1 and S2.

**FIG. 4.**
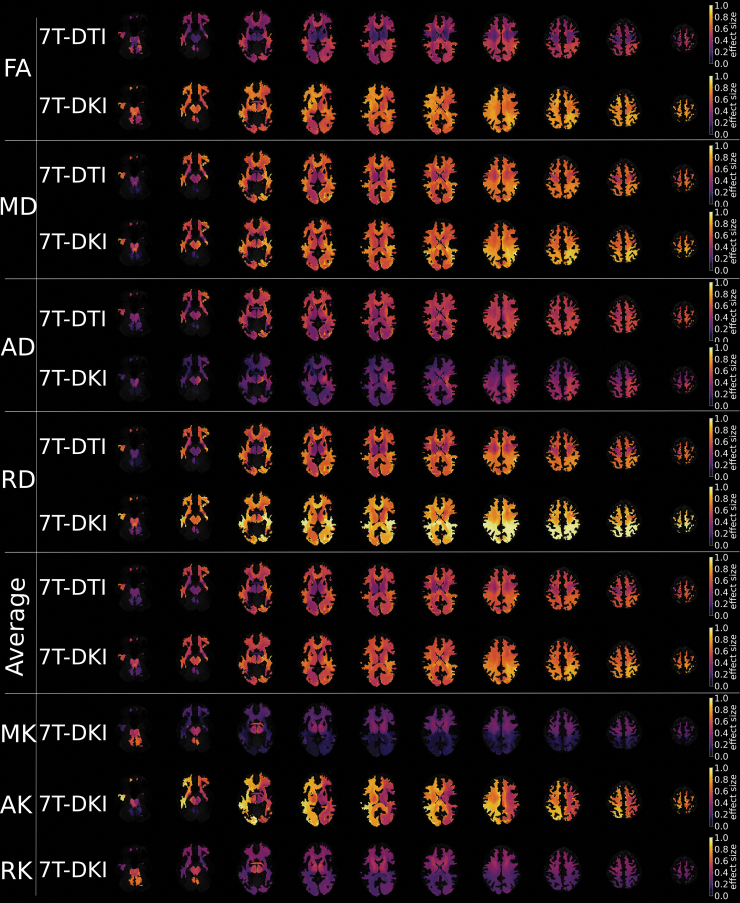
Effect sizes of diffusion parameters. Effect sizes, plotted in axial slices of the brain, for differences between sports-related concussion (SRC) athletes and controls in the metrics of diffusion tensor imaging (DTI) and diffusion kurtosis imaging (DKI). Included metrics are fractional anisotropy (FA), mean diffusivity (MD), axial diffusivity (AD), and radial diffusivity (RD) for DTI, and FA, MD, AD, RD, mean kurtosis (MK), axial kurtosis (AK), and radial kurtosis (RK) for DKI. The two “average” rows depict the average result for DTI and DKI across FA, MD, AD, and RD. Dark colors represent a low effect size and yellow represents a high effect size.

These results were further strengthened by a permutation analysis, which showed that the number of significant tracts between SRC athletes and controls exceeded the number of significant tracts under the null hypothesis for DKI FA, MD, RD, AK, and RK (Table 4). The alterations were widespread in all areas of WM (Fig. 4). There were no significant differences in WM volumes between SRC athletes and controls analyzed with DTI and DKI TractSeg (*p* > 0.05).

**Table 4.** Permutation Analysis

	ɑ = 0.05			ɑ = 0.01		
	*T*	*p*	95th	*T*	*p*	95th
DTI FA	3	n.s.	16	0	n.s.	3
DTI MD	18	0.047	18	2	n.s.	3
DTI AD	14	n.s.	16	2	n.s.	3
DTI RD	11	n.s.	17	1	n.s.	3
**DKI FA**	**51**	**0.0001**	16	**33**	**0.005**	4
**DKI MD**	**19**	**0.036**	17	5	n.s.	3
DKI AD	0	n.s.	15	0	n.s.	3
**DKI RD**	**30**	**0.006**	17	**13**	**0.006**	3
DKI MK	6	n.s.	20	0	n.s.	2
**DKI AK**	**35**	**0.019**	20	**16**	**0.009**	3
**DKI RK**	**28**	**0.036**	22	2	n.s.	2

Columns show the name of the magnetic resonance imaging (MRI) metric with diffusion tensor imaging (DTI) and diffusion kurtosis imaging (DKI). The permutation analysis includes 20 sports-related concussion (SRC) athletes and 21 controls for DTI, and 19 SRC athletes and 20 controls for DKI. DTI metrics are fractional anisotropy (FA), mean diffusivity (MD), axial diffusivity (AD), and radial diffusivity (RD), and DKI metrics are FA, MD, AD, RD, mean kurtosis (MK), axial kurtosis (AK), and radial kurtosis (RK). Three properties, the number of significant tracts (#T), *p* value from the permutation testing of the number of significant tracts expected under the null hypothesis, and the 95th percentile of the number of tracts that shows significant differences under the null hypothesis (i.e., the highest number of false positives expected), are shown for two different significance thresholds in the per-tract *U* tests, an alpha (ɑ) set to 0.05 and an ɑ set to 0.01. Significantly differing tracts are bolded: tracts with *p* values under the set ɑ and with number of differing tracts over the expected for the 95th percentile.

### CSF biomarkers

Sixteen SRC athletes consented to CSF sampling. SRC athletes had a median NfL of 219 ng/L (IQR 153–279) and a GFAP of 248 ng/L (IQR 194–299).

### Correlation analyses

Correlations were analyzed for the SRC athletes only (Table 5). The number of SRCs and SCAT5 data did not correlate significantly with the psychometric neuropsychology tests, with brain volumes or with DTI and DKI metrics averaged across all tracts (*p* > 0.05).

**Table 5.** Correlation Analysis

r_s_ (*p *value)		RBANS Whole Scale	SDMT	d2	MFS	BRIEF Whole Scale	BRIEF Working Memory	HADS Anxiety	HADS Depression	NfL, ng/L	GFAP, ng/L
DTI	FA	-0.150 (0.527)	0.030 (0.900)	0.156 (0.510)	-0.021 (0.930)	-0.005 (0.982)	0.299 (0.200)	-0.032 (0.930)	-0.142 (0.550)	0.397 (0.143)	-0.202 (0.470)
	MD	0.242 (0.305)	0.027 (0.910)	-0.007 (0.977)	-0.002 (0.995)	0.168 (0.480)	-0.301 (0.198)	**0.268 (0.253)**	**0.335 (0.149)**	**-0.620 (0.014)**	0.093 (0.742)
	AD	0.248 (0.291)	0.118 (0.619)	-0.007 (0.977)	-0.093 (0.698)	0.153 (0.518)	-0.285 (0.224)	**0.285 (0.223)**	**0.361 (0.118)**	**-0.542 (0.037)**	0.173 (0.537)
	RD	0.218 (0.355)	0.086 (0.719)	0.040 (0.867)	-0.102 (0.668)	0.024 (0.920)	-0.418 (0.067)	**0.201 (0.396)**	**0.243 (0.302)**	**-0.558 (0.031)**	0.057 (0.840)
DKI	FA	0.163 (0.504)	-0.021 (0.932)	0.232 (0.340)	0.057 (0.817)	0.187 (0.444)	0.351 (0.140)	**-0.448 (0.054)**	**0.128 (0.602)**	**0.664 (0.010)**	0.198 (0.497)
	MD	0.119 (0.626)	0.127 (0.606)	0.015 (0.952)	-0.022 (0.929)	0.089 (0.718)	-0.269 (0.265)	**0.269 (0.266)**	**0.156 (0.523)**	**-0.583 (0.029)**	-0.139 (0.637)
	AD	0.107 (0.663)	0.180 (0.460)	0.071 (0.772)	-0.114 (0.642)	0.024 (0.923)	-0.253 (0.296)	0.156 (0.523)	0.071 (0.771)	-0.431 (0.124)	-0.227 (0.436)
	RD	0.042 (0.864)	0.048 (0.844)	-0.039 (0.872)	0.025 (0.920)	0.123 (0.616)	-0.252 (0.297)	**0.324 (0.176)**	**0.196 (0.422)**	**-0.601 (0.023)**	-0.156 (0.594)
	MK	-0.125 (0.611)	0.069 (0.778)	-0.198 (0.416)	-0.108 (0.660)	0.003 (0.991)	0.064 (0.793)	0.189 (0.438)	-0.154 (0.528)	-0.271 (0.349)	-0.288 (0.318)
	AK	0.066 (0.789)	-0.046 (0.853)	-0.062 (0.800)	0.370 (0.119)	0.125 (0.609)	0.348 (0.145)	-0.283 (0.240)	-0.268 (0.267)	0.429 (0.126)	0.009 (0.976)
	RK	-0.122 (0.619)	0.110 (0.654)	-0.121 (0.621)	-0.165 (0.500)	-0.014 (0.955)	0.017 (0.946)	0.199 (0.415)	-0.142 (0.562)	-0.363 (0.202)	-0.317 (0.270)

Spearman's correlation (r_s_) coefficient and *p* values for the correlations of neuropsychology tests (raw scores) and biomarkers with single white matter region of interest (WM ROI) values for the diffusion tensor imaging (DTI) and diffusion kurtosis imaging (DKI) metrics. Included metrics for correlations are fractional anisotropy (FA), mean diffusivity (MD), axial diffusivity (AD), and radial diffusivity (RD) for DTI, and FA, MD, AD, RD, mean kurtosis (MK), axial kurtosis (AK), and radial kurtosis (RK) for DKI. Included neuropsychology tests are the Repeatable Battery for the Assessment of Neuropsychological Status (RBANS), the d2 test of attention (d2), Symbol Digit Modalities Test (SDMT), the Behaviour Rating Inventory of Executive Function-Adult version (BRIEF-A) whole scale and working memory, and the Hospital Anxiety and Depression Scale (HADS) anxiety and depression. Biomarkers include neurofilament light (NfL) and glial fibrillary acidic protein (GFAP). There were no significant correlations between neuropsychology tests and DTI or DKI metrics; however, NfL correlated significantly with several DTI and DKI metrics, shown in bold. Included SRC athletes in the correlation analyses are for DTI; RBANS 20, SDMT 20, d2 20, Mental Fatigue Scale (MFS) 20, BRIEF whole scale 20, BRIEF working memory 20, HADS anxiety 20, HADS depression 20, NfL 15, and GFAP 15, and for DKI; RBANS 19, SDMT 19, d2 19, MFS 19, BRIEF whole scale 19, BRIEF working memory 19, HADS anxiety 19, HADS depression 19, NfL 14, and GFAP 14.

To evaluate correlations between global measures of cognition and impaired diffusion metrics RBANS whole scale, SDMT, d2, MFS, BRIEF whole scale, and BRIEF working memory were correlated to the single WM ROIs for each of the DTI and DKI metrics. No significant correlations were found (see Table 5). BRIEF behavioral regulation index correlated significantly with DKI MD (r_s_ = 0.499, *p* = 0.030) and DKI RD (r_s_ = 0.573, *p =* 0.010) in the left cingulum. To test for a relationship between the hippocampus and episodic memory we correlated hippocampal volume to RBANS list learning and list recall, which were not significant (*p =* 0.611 and *p =* 0.561, respectively).

**FIG. 5.**
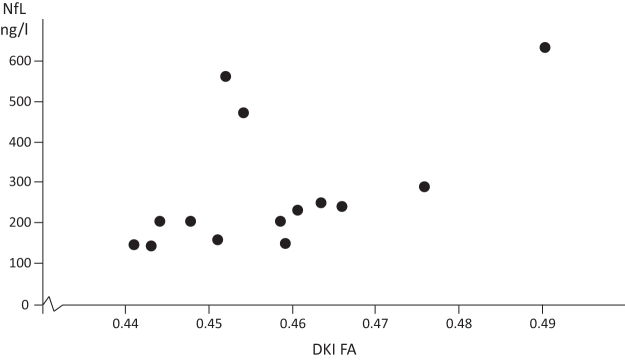
Correlation between diffusion kurtosis imaging (DKI) fractional anisotropy (FA) and cerebrospinal fluid (CSF) levels of neurofilament light (NfL). Single correlation values for DKI FA and NfL. Fourteen sports-related concussion (SRC) athletes were included in the analysis.

NfL correlated negatively with single WM ROI for DTI MD (r_s_ = −0.620, *p* = 0.014), DTI AD (r_s_ = −0.542, *p* = 0.037), DTI RD (r_s_ = – 0.558, *p* = 0.031), DKI MD (r_s_ = −0.583, *p* = 0.029) and DKI RD (r_s_ = −0.601, *p* = 0.023), and positively with single WM ROI for DKI FA (r_s_ = 0.664, *p =* 0.010, Fig. 5). GFAP did not correlate significantly with any DTI or DKI metrics. Biomarkers in CSF did not correlate with SCAT5 or the neuropsychology tests, with the exception of NfL and the BRIEF behavioral regulation index (r_s_ = -0.704, *p =* 0.002, data not shown).

## Discussion

The present study investigated neurocognitive functioning, structural abnormalities in GM and WM, and CSF biomarkers in a well-characterized group of athletes with long-lasting PPCS. Our main findings were that despite having no gross findings on routine clinical neuroimaging, SRC athletes had marked deficits on neuropsychological functioning and showed evidence of widespread structural changes in a large proportion of WM tracts when studied by 7T DKI. Further, the axonal injury marker NfL correlated with several of the diffusion metrics. These findings indicate that advanced neuroimaging and CSF biomarkers are needed to explore the extent of WM abnormalities following SRC.

The SRC athletes had sustained a median of five SRCs, reported a high symptom burden at the time of investigation, and would meet the ICD-10 criteria for post-concussion syndrome. The symptoms included elevated ratings of depression, anxiety, and mental fatigue; subjective experiences of executive dysfunction; and impaired life satisfaction. The enrollment of the PPCS cohort could, as a result of self-selection, have introduced a bias with symptoms unrelated to SRC-induced factors but instead caused by mental health concerns. We regard this as unlikely because no elite athlete had any pre-existing mental health issues and the PPCS were continuous, without symptom-free periods post-concussion. Moreover, the causes of PPCS may be multifactorial, and psychological processes and pre-injury factors could contribute.^69^ We argue that biological processes are indeed present at prolonged times post-injury, supported by recent findings from our group.^70^

Our results revealed deficits in neuropsychological functioning, particularly in the domains of immediate memory, verbal abilities, and attentional functions. Compared with controls, SRC athletes performed significantly worse on all psychometric measures except the Digit span working memory test. Several factors other than SRCs can influence perceived mental health, such as SRC-induced post-traumatic stress and not being able to continue sport participation or attend work, studies, or social events to the same extent as previously. Although other factors may contribute to mental health issues, given the extent of structural abnormalities on 7T DKI, we argue that the observed WM abnormalities may give rise to neurobiologically based dysregulation of emotional homeostasis following SRC.^71^

The microstructural alterations detected with 7T DKI in this group of symptomatic SRC athletes at an average time of 2.6 years since their last SRC suggest persisting, or even evolving neuropathology in the WM, as supported by our CSF NfL data. At the time of SRC, rotational forces cause tension in WM tracts, and this axonal stretch may lead to axonal beading,^72^ which may explain increased AK.^15,73^ Axonal beading is the accumulation of varicosities along the axis of an axon, which may be caused by a tension-driven destabilization of microtubules.^74^ The theory of axonal beading is further strengthened and argued for in an animal model of mTBI, where an increase in AK and MK was seen.^75^ Neurodegenerative processes tend to yield reduced FA. However, increased FA in SRC as observed in our present study has been observed previously. In our study we observed increased DKI FA and AK, and decreased RD and RK, which may be evidence of axonal beading, gliosis, and astrogliosis supported by a post-mortem study of athletes with previous SRCs.^76^ Other mechanisms such as Wallerian degeneration, a concomitant inflammatory response, and SRC-induced neurodegeneration may also contribute.^77–79^ However, this needs verification by histopathological evaluation. The correlation between NfL and several DTI and DKI metrics argues for an ongoing axonal injury process rather than astrogliosis as an explanation of the altered diffusion metrics. Recently, serum NfL and GFAP levels were evaluated over several years following injury,^30^ and whereas NfL and GFAP correlated with WM atrophy and DTI measures of axonal injury, the prognostic utility of GFAP was inferior to that of NfL.^30^ The correlations between diffusion metrics and the axonal injury marker NfL indicate a progressive axonal injury process. The microstructural changes found in this study were widespread in the WM and present in long tracts, central structures, and in both hemispheres, and were mainly supratentorial. In previous studies, a general pattern of abnormalities was observed,^13,15^ which was expected in view of the rotational forces causing global WM tract shearing.

In the diffusion analysis, DKI yielded higher effect sizes than DTI (see the “average rows” in Fig. 4). Diffusion MRI (DTI or DKI) metrics characterize alternations in WM morphology, described by water diffusivity within the WM tracts. Our results indicate that DKI is more sensitive in the evaluation of PPCS athletes than DTI; however, the merit of DKI over DTI can only be appreciated when both protocols are implemented equally. DKI is a more demanding protocol than DTI (multi-shell acquisition, higher b-values, necessitates diffusion encoding with longer gradient pulses), and therefore, DKI is performed with longer echo times than DTI, which reduces the baseline SNR ratio. This is often compensated for by reducing the spatial resolution in DKI compared with DTI. In such a case, DKI might not demonstrate any additive values compared with DTI, and to date we cannot firmly argue that DKI should replace DTI in clinical routine. A different observation when comparing DTI with DKI was reported in a study on SRC athletes, in which MD differed from DTI more extensively across the WM skeleton than any DKI parameter.^80^ A possible explanation is that the DTI protocol featured fewer diffusion encodings than the DKI protocol (36 and 52, respectively), which therefore leads to a slight disadvantage in terms of protocol performance. However, previous studies investigating what effect the number of gradient directions has on the accuracy of direction-sensitive diffusion parameters such as FA, AD, and RD showed that above a certain number of directions (∼ 25) the accuracy does not improve substantially from an increased number of encoding directions.^81^

Despite the high sensitivity of DTI and DKI to detect signs of microstructural alterations in SRC, there is considerable ambiguity between studies, showing both increases and decreases in the same metrics.^12–18,20–23,73^ Some of the ambiguity can probably be explained by the diversity in which metrics were acquired and analyzed and at which post-injury time points and in which populations they were evaluated. DTI and DKI metrics change over time with the pathology, and studies indicate that metrics can evolve over time, or return to normal levels.^22,23,27,28^ The modalities differ in sensitivity,^15,18,21,73^ and can be sensitive to different changes at different time points.^17,23^ Most SRC studies have a male dominance or only include men,^13–15^ and there may be important sex differences. In a previous mTBI study, significantly different DTI metrics compared with controls were only observed in symptomatic patients and not in the asymptomatic group.^16^ Similarly, a study of DTI and DKI following mTBI revealed differences to controls at 72 h and 3 months, and when stratifying based on symptoms at 3 months, symptomatic patients had signs of pathology not present in the asymptomatic group.^17^ In another study, the risk of developing PPCS could be predicted using DTI and DKI metrics at 72 h post-injury.^18^ Further, microstructural alterations have been shown to extend beyond symptom resolution in SRC athletes.^13,15^ This is evidence of a high sensitivity to microstructural change following SRC, with impairments observed even in symptom-free athletes, and more impairments observed in athletes with prolonged symptoms. DTI and DKI, with the addition of NfL, may therefore aid in determining the neurobiological correlates, and which athletes may be at risk of prolonged symptoms.

### Limitations

The present study evaluates a rather small, yet highly characterized cohort of SRC athletes with PPCS ≥6 months. However, this cohort may not be representative of all SRC athletes. The discrepancies in cognitive performances between SRC athletes and our control group may have been enhanced by factors other than SRCs. Although matched by age and sex, the controls had more years of education, which is related to better psychometric performance.^82^ Still, the majority of SRC athletes in the present study demonstrated cognitive difficulties by deviating from test-specific norms. The level of education in the SRC group is consistent with the mean years of education in the United States and Sweden – from where norms are obtained. The PPCS severity may have contributed to impaired ability for higher education and therefore have contributed to the lower level of education observed in our athlete cohort.

Although CSF sampling in PPCS athletes is rare, we acknowledge the limitations caused by the small sample size and the fact that CSF sampling was not conducted in our control cohort (it was not permitted by the ethical committee). Therefore, our study may lack statistical power in detecting correlations using the CSF biomarker findings. It should be noted that our included cohort displayed a very high symptom severity and number of symptoms, although as SCAT symptoms were not obtained from our control cohort, direct comparisons cannot be made. T1-weighed imaging, DTI, and DKI may have benefited from the increased SNR at 7T. However, T1-weighted imaging at 7T is known to occasionally suffer from increased B1+ inhomogeneity with artefacts in low signal intensity regions, mainly in the temporal lobes.^83^ Such specific artefacts were present in two athletes and three controls in our study, which led to their exclusion from the volumetric analysis.

Multiple comparisons, not corrected for, may lead to a risk of statistical type II error. We acknowledge that the absence of such corrections may increase the risk of type II errors, although we argue against the differences being merely caused by false positives. First, in this exploratory study, we performed a permutation analysis that showed that the number of tracts showing significant differences between SRC athletes and controls were larger than expected from chance alone for many of the DKI parameters. Second, we introduced the single WM ROI, which also displayed significant differences across the groups. Regardless, a larger PPCS cohort could have enabled stronger conclusions, although this would have been difficult in view of the narrow inclusion criteria.

## Conclusion

In this study, persistent symptoms following SRC were associated with several neurocognitive impairments as well as signs of depression, anxiety, mental fatigue, executive dysfunction, and impaired life satisfaction. We found evidence of widespread WM microstructural alterations on DKI. The correlation of WM abnormalities on DKI and CSF NfL levels, but not GFAP, points toward an important role for an ongoing axonal injury process in PPCS athletes.

## Transparency, Rigor, and Reproducibility Summary

The study and the analysis plan were not formally pre-registered, although the analysis plan was pre-specified in the study planning. A sample size of 40–50 subjects, SRC athletes and controls equally distributed, was planned based on the limited availability of Swedish SRC athletes meeting the strict inclusion criteria. The included sample size was 22 subjects per group (please see Table S3 for further details). The licensed neuropsychologist conducting the assessments was aware of the clinical status of the subjects (SRC athletes or healthy controls) but blinded to the neuroimaging and CSF biomarkers results. Participants were unable to guess the results of their assessments, as the results were analyzed after their last clinical evaluation, whereafter the participants were informed about their neuropsychology results and provided with recommendations when appropriate. Data collection and processing of MRI data were performed by investigators unaware of relevant participants' characteristics (medical history, neuropsychology, and lumbar puncture). Data were labeled using codes linked to participant identifying information.

Because of the limited sample size and particular patient features, making it impossible to blind the data, the data analysis (statistics) was performed by investigators who were aware of relevant characteristics of the participants. Data were acquired between June 2018 and June 2020, Monday to Fridays between 7:30 and 16:30. Data were collected with subjects well rested, starting with neuropsychology, followed by MRI, with the lumbar puncture being the last procedure during the day. All data sets were analyzed at the same time using the Statistical Package for the Social Sciences (SPSS Inc., Version 25, IBM, New York) with methods specified in the Methods section. Specific equipment and software used to perform acquisition and analysis are widely available or may be available upon request from each specific company and The National 7T facility at Lund University Bioimaging. The key inclusion criteria for PPCS varies in the literature^2^ although they are typically, as in our present study, based on symptom duration and severity. The primary clinical outcome measures (PPCS symptoms, neuropsychology, DTI/DKI metrics, and blood or, as used here, CSF biomarkers) are increasingly used in this research field but may not be widely established as standards. The statistical tests used were based on the testing for normality/non-normality and sample size. Statistical analysis and review were performed by the authors (A.G., M.N., N.M.) with qualifications including specific statistical training and expertise. Correction for multiple comparison was not performed, which is described in detail in the Statistical analysis and Discussion sections. Replication by the study group and external validation are ongoing. De-identified data from this study are not available in a public archive but may be received from the authors upon reasonable request. Analytical codes used to conduct the analyses presented in this study are not available in a public repository.
