# Supplementary material for: Widespread White Matter Abnormalities in Concussed Athletes Detected by 7T Diffusion Magnetic Resonance Imaging
Source: J Neurotrauma. 2024 Jul 17;41(13-14):1533–49. doi: 10.1089/neu.2023.0099 (PMC11564857; doi:10.1089/neu.2023.0099)
Supplement: Supplementary Table S1 [file neu.2023.0099_suppl_table1.pdf]

Supplemental Table 1

**Table 1S. Diffusion *P*-values**										
	DTI				DKI					
	FA	MD	AD	RD	FA	MD	AD	RD	MK	AK
Arcuate fascicle left	+	-	-	-	+	-	+	-	-	+
Arcuate fascicle right	+	-	-	-	+	-	+	-	-	+
Anterior Thalamic Radiation left	-	-	-	+	+	-	+	-	-	+
Anterior Thalamic Radiation right	-	-	-	+	+	+	+	+	-	+
Commissure Anterior	+	-	-	-	+	-	-	-	-	+
Rostrum	+	-	-	-	+	-	-	-	-	+
Genu	+	-	-	-	+	-	+	-	-	+
Rostral body (Premotor)	-	+	-	-	+	-	-	-	-	+
Anterior midbody (Primary Motor)	-	-	-	+	+	-	-	+	-	+
Posterior midbody (Primary Somatosensory)	+	-	-	-	+	-	+	-	-	+
Isthmus	+	-	-	-	+	-	+	-	-	+
Splenium	+	-	+	-	+	-	+	-	-	+
Cingulum left	+	-	-	-	+	-	-	-	-	+
Cingulum right	+	-	-	-	+	-	+	-	-	+
Corticospinal tract left	-	-	-	-	+	-	-	-	-	+
Corticospinal tract	-	-	-	+	+	-	-	-	-	+

right										
Middle longitudinal fascicle left	+	-	-	-	+	-	-	-	-	+
Middle longitudinal fascicle right	+	-	-	-	+	-	-	-	-	+
Fronto-pontine tract left	+	-	+	+	+	-	-	-	-	+
Fronto-pontine tract right	+	-	+	-	+	-	-	-	-	+
Fornix left	+	+	+	+	+	+	-	+	-	+
Fornix right	-	+	+	+	-	-	-	-	-	+
Inferior cerebellar peduncle left	-	+	+	+	+	-	-	-	-	+
Inferior cerebellar peduncle right	-	+	+	-	+	+	+	-	-	-
Inferior occipito-frontal fascicle left	+	-	-	-	+	-	-	-	-	+
Inferior occipito-frontal fascicle right	+	-	-	-	+	-	-	-	-	+
Inferior longitudinal fascicle left	+	-	-	-	+	-	+	-	-	+
Inferior longitudinal fascicle right	+	-	-	-	+	-	+	-	-	+
Middle cerebellar peduncle	-	-	-	-	+	-	-	-	-	-
Optic radiation	+	+	+	-	+	-	+	-	-	+

left										
Optic radiation right	+	-	-	-	+	-	+	-	-	+
Parieto occipital pontine left	-	-	-	-	+	-	-	-	-	+
Parieto occipital pontine right	+	-	-	-	+	-	-	-	-	+
Superior cerebellar peduncle left	-	-	-	-	+	-	-	-	-	-
Superior cerebellar peduncle right	-	-	-	-	+	-	+	-	-	-
Superior longitudinal fascicle I left	-	-	-	-	+	-	-	-	-	+
Superior longitudinal fascicle I right	+	-	-	-	+	-	-	-	-	+
Superior longitudinal fascicle II left	+	-	-	-	+	-	+	-	-	+
Superior longitudinal fascicle II right	+	-	-	-	+	-	-	-	-	+
Superior longitudinal fascicle III left	+	-	-	-	+	-	-	-	-	+
Superior longitudinal fascicle III right	+	-	+	-	+	+	-	-	-	+
Superior Thalamic Radiation left	-	+	-	+	+	-	-	-	-	+
Superior	+	+	-	+	+	-	-	-	-	+

Thalamic Radiation right										
Uncinate fascicle left	-	-	-	-	+	-	+	-	-	+
Uncinate fascicle right	-	-	-	-	+	-	+	-	-	+
Corpus Callosum	+	-	-	-	+	-	+	-	-	+
Thalamo-prefrontal left	+	-	-	-	+	-	+	-	-	+
Thalamo-prefrontal right	+	-	-	-	+	-	+	-	-	+
Thalamo-premotor left	+	-	-	+	+	-	-	-	-	+
Thalamo-premotor right	+	-	-	-	+	-	+	-	-	+
Thalamo-precentral left	-	-	-	+	+	-	-	-	-	+
Thalamo-precentral right	+	-	-	+	+	-	+	-	-	+
Thalamo-postcentral left	-	-	-	-	+	-	+	-	-	+
Thalamo-postcentral right	+	-	-	-	+	-	-	-	-	+
Thalamo-parietal left	+	-	-	-	+	-	-	-	-	+
Thalamo-parietal right	+	-	-	-	+	-	-	-	-	+
Thalamo-occipital left	+	+	+	-	+	-	+	-	-	+
Thalamo-occipital right	+	-	-	-	+	-	+	-	-	+
Striato-fronto-orbital left	+	-	-	+	+	-	-	-	-	+

Striato-fronto-orbital right	+	-	-	-	+	+	+	-	-	+
Striato-prefrontal left	+	-	-	-	+	-	+	-	-	+
Striato-prefrontal right	+	-	-	-	+	-	+	-	-	+
Striato-premotor left	+	-	-	-	+	-	-	-	-	+
Striato-premotor right	-	-	-	-	+	-	-	-	-	+
Striato-precentral left	+	-	-	-	+	-	-	-	-	+
Striato-precentral right	-	-	-	-	+	-	-	-	-	+
Striato-postcentral left	+	-	-	-	+	-	+	-	-	+
Striato-postcentral right	-	-	-	-	+	-	-	-	-	+
Striato-parietal left	+	-	-	-	+	-	-	-	-	+
Striato-parietal right	+	-	-	-	+	-	-	-	-	+
Striato-occipital left	+	-	+	-	+	-	+	-	-	+
Striato-occipital right	+	-	-	-	+	-	+	-	-	+
Global WM metric value (p-value)	+ (0.322)	- (0.335)	- (0.389)	- (0.262)	+ (< 0.001)	- (0.144)	+ (0.822)	- (0.011)	- (0.053)	+ (0.023)
Percent of differing structures	4.2%	25%	19%	17%	72%	26%	0%	50%	8.3%	49%

Supplemental Table 1. Diffusion *P*-values

The p-values of the comparison between SRC athletes (DTI n=20 and DKI n=19) and controls (DTI n= 21 and DKI n=20) of the 72 anatomical tracts and one global white matter (WM) metric value, for the investigated metrics with diffusion tensor imaging (DTI) and diffusion kurtosis imaging (DKI). DTI metrics are fractional anisotropy (FA), mean diffusivity (MD), axial diffusivity (AD) and radial diffusivity (RD), and DKI metrics are FA, MD, AD, RD, mean kurtosis (MK), axial kurtosis (AK) and radial kurtosis (RK). If SRC athletes had a higher metric compared with the controls it is marked with "+", and if they had a lower metric it is marked with "-". Significant P-values are highlighted in a grey colour.
