# Supplementary material for: Widespread White Matter Abnormalities in Concussed Athletes Detected by 7T Diffusion Magnetic Resonance Imaging
Source: J Neurotrauma. 2024 Jul 17;41(13-14):1533–49. doi: 10.1089/neu.2023.0099 (PMC11564857; doi:10.1089/neu.2023.0099)
Supplement: Supplementary Table S2 [file neu.2023.0099_suppl_table2.pdf]

Supplemental Table 2

A. DTI - FA		
	SRC athletes, median (IQR) *n*=20	Controls, median (IQR) *n*=21
Arcuate_fascicle_left	0.391 (0.382-0.407)	0.386 (0.369-0.407)
Arcuate_fascicle_right	0.378 (0.372-0.389)	0.370 (0.360-0.384)
Anterior_Thalamic_Radiation_left	0.352 (0.339-0.368)	0.355 (0.339-0.366)
Anterior_Thalamic_Radiation_right	0.346 (0.336-0.360)	0.347 (0.334-0.356)
Commissure_Anterior	0.376 (0.343-0.389)	0.355 (0.323-0.399)
Rostrum	0.482 (0.453-0.499)	0.466 (0.450-0.482)
Genu	0.384 (0.366-0.392)	0.374 (0.355-0.386)
Rostral_body_(Premotor)	0.424 (0.418-0.447)	0.427 (0.410-0.443)
Anterior_midbody_(Primary_Motor)	0.451 (0.438-0.467)	0.453 (0.436-0.461)
Posterior_midbody_(Primary_Somatosensory)	0.444 (0.429-0.455)	0.441 (0.417-0.463)
Isthmus	0.412 (0.403-0.430)	0.403 (0.389-0.430)
Splenium	0.427 (0.410-0.454)	0.417 (0.400-0.446)
Cingulum_left	0.389 (0.381-0.405)	0.380 (0.364-0.402)
Cingulum_right	0.387 (0.374-0.392)	0.371 (0.355-0.397)
Corticospinal_tract_left	0.496 (0.478-0.503)	0.497 (0.482-0.514)
Corticospinal_tract_right	0.490 (0.387-0.508)	0.498 (0.480-0.509)
Middle_longitudinal_fascicle_left	0.402 (0.387-0.413)	0.387 (0.373-0.412)
**Middle_longitudinal_fascicle_right**	**0.402 (0.389-0.411)**	**0.376 (0.370-0.408)**
Fronto-pontine_tract_left	0.454 (0.456-0.469)	0.448 (0.428-0.463)
Fronto-pontine_tract_right	0.448 (0.433-0.461)	0.444 (0.431-0.460)
Fornix_left	0.419 (0.395-0.451)	0.410 (0.385-0.436)
Fornix_right	0.387 (0.366-0.430)	0.407 (0.360-0.431)
Inferior_cerebellar_peduncle_left	0.438 (0.403-0.474)	0.460 (0.415-0.482)
Inferior_cerebellar_peduncle_right	0.415 (0.402-0.450)	0.424 (0.393-0.452)
Inferior_occipito-frontal_fascicle_left	0.375 (0.361-0.382)	0.364 (0.354-0.375)
Inferior_occipito-frontal_fascicle_right	0.356 (0.344-0.369)	0.345 (0.338-0.358)
Inferior_longitudinal_fascicle_left	0.427 (0.418-0.453)	0.417 (0.409-0.442)
Inferior_longitudinal_fascicle_right	0.416 (0.395-0.456)	0.410 (0.390-0.428)
Middle_cerebellar_peduncle	0.467 (0.450-0.487)	0.488 (0.470-0.498)
Optic_radiation_left	0.418 (0.406-0.442)	0.412 (0.398-0.428)
Optic_radiation_right	0.423 (0.408-0.439)	0.408 (0.403-0.420)
Parieto_occipital_pontine_left	0.446 (0.446-0.459)	0.448 (0.428-0.464)
Parieto_occipital_pontine_right	0.458 (0.447-0.470)	0.452 (0.437-0.470)
Superior_cerebellar_peduncle_left	0.426 (0.411-0.441)	0.437 (0.416-0.462)
Superior_cerebellar_peduncle_right	0.425 (0.408-0.444)	0.433 (0.417-0.455)
Superior_longitudinal_fascicle_I_left	0.438 (0.426-0.454)	0.439 (0.408-0.449)
Superior_longitudinal_fascicle_I_right	0.426 (0.414-0.437)	0.419 (0.400-0.434)
Superior_longitudinal_fascicle_II_left	0.416 (0.402-0.427)	0.411 (0.389-0.428)
**Superior_longitudinal_fascicle_II_right**	**0.399 (0.383-0.407)**	**0.380 (0.374-0.393)**
Superior_longitudinal_fascicle_III_left	0.415 (0.405-0.431)	0.399 (0.388-0.419)
Superior_longitudinal_fascicle_III_right	0.389 (0.378-0.398)	0.381 (0.370-0.392)

Superior_Thalamic_Radiation_left	0.452 (0.440-0.469)	0.455 (0.430-0.469)
Superior_Thalamic_Radiation_right	0.460 (0.436-0.467)	0.453 (0.436-0.474)
Uncinate_fascicle_left	0.394 (0.380-0.415)	0.396 (0.378-0.409)
Uncinate_fascicle_right	0.377 (0.357-0.397)	0.378 (0.353-0.401)
Corpus_Callosum-all	0.385 (0.377-0.403)	0.380 (0.365-0.399)
Thalamo-prefrontal_left	0.382 (0.369-0.395)	0.377 (0.356-0.387)
Thalamo-prefrontal_right	0.368 (0.355-0.380)	0.360 (0.350-0.377)
Thalamo-premotor_left	0.402 (0.390-0.409)	0.400 (0.386-0.411)
Thalamo-premotor_right	0.396 (0.385-0.409)	0.395 (0.379-0.406)
Thalamo-precentral_left	0.430 (0.421-0.444)	0.437 (0.421-0.440)
Thalamo-precentral_right	0.423 (0.413-0.436)	0.421 (0.413-0.438)
Thalamo-postcentral_left	0.418 (0.403-0.431)	0.420 (0.391-0.426)
Thalamo-postcentral_right	0.427 (0.415-0.438)	0.423 (0.405-0.442)
Thalamo-parietal_left	0.406 (0.388-0.419)	0.397 (0.382-0.418)
Thalamo-parietal_right	0.409 (0.399-0.424)	0.405 (0.387-0.421)
Thalamo-occipital_left	0.412 (0.397-0.434)	0.403 (0.393-0.420)
Thalamo-occipital_right	0.412 (0.396-0.428)	0.397 (0.390-0.409)
Striato-fronto-orbital_left	0.387 (0.371-0.398)	0.382 (0.372-0.399)
Striato-fronto-orbital_right	0.415 (0.398-0.425)	0.403 (0.386-0.423)
Striato-prefrontal_left	0.370 (0.355-0.379)	0.363 (0.345-0.369)
Striato-prefrontal_right	0.366 (0.355-0.379)	0.360 (0.348-0.377)
Striato-premotor_left	0.389 (0.378-0.398)	0.386 (0.372-0.403)
Striato-premotor_right	0.396 (0.386-0.403)	0.389 (0.376-0.408)
Striato-precentral_left	0.423 (0.408-0.430)	0.420 (0.399-0.433)
Striato-precentral_right	0.420 (0.409-0.428)	0.423 (0.412-0.431)
Striato-postcentral_left	0.408 (0.391-0.416)	0.402 (0.382-0.420)
Striato-postcentral_right	0.415 (0.410-0.429)	0.419 (0.402-0.436)
Striato-parietal_left	0.403 (0.383-0.414)	0.392 (0.379-0.413)
Striato-parietal_right	0.411 (0.400-0.424)	0.402 (0.390-0.420)
Striato-occipital_left	0.424 (0.410-0.448)	0.410 (0.403-0.423)
**Striato-occipital_right**	**0.413 (0.390-0.425)**	**0.396 (0.389-0.404)**
Global WM metric value	0.414 (0.403-0.421)	0.412 (0.397-0.420)

B. DTI - MD

	SRC athletes, median (IQR) *n*=20	Controls, median (IQR) *n*=21
**Arcuate_fascicle_left**	**0.720 (0.706-0.732)**	**0.729 (0.718-0.745)**
Arcuate_fascicle_right	0.727 (0.717-0.741)	0.734 (0.723-0.755)
Anterior_Thalamic_Radiation_left	0.769 (0.758-0.806)	0.774 (0.758-0.791)
Anterior_Thalamic_Radiation_right	0.774 (0.758-0.792)	0.774 (0.757-0.814)
Commissure_Anterior	0.842 (0.819-0.903)	0.871 (0.831-0.889)
Rostrum	0.815 (0.783-0.835)	0.834 (0.801-0.877)
Genu	0.807 (0.782-0.816)	0.816 (0.798-0.843)
Rostral_body_(Premotor)	0.800 (0.768-0.805)	0.799 (0.777-0.828)
Anterior_midbody_(Primary_Motor)	0.764 (0.736-0.781)	0.766 (0.751-0.787)
Posterior_midbody_(Primary_Somatosensory)	0.793 (0.760-0.798)	0.802 (0.775-0.815)
Isthmus	0.790 (0.768-0.817)	0.802 (0.788-0.839)

Splenium	0.821 (0.789-0.851)	0.838 (0.796-0.862)
**Cingulum_left**	**0.742 (0.726-0.746)**	**0.759 (0.741-0.769)**
**Cingulum_right**	**0.732 (0.725-0.745)**	**0.748 (0.732-0.766)**
Corticospinal_tract_left	0.705 (0.694-0.718)	0.709 (0.698-0.717)
Corticospinal_tract_right	0.699 (0.693-0.712)	0.699 (0.689-0.714)
**Middle_longitudinal_fascicle_left**	**0.741 (0.729-0.751)**	**0.762 (0.748-0.781)**
**Middle_longitudinal_fascicle_right**	**0.752 (0.735-0.761)**	**0.773 (0.753-0.789)**
Fronto-pontine_tract_left	0.733 (0.724-0.743)	0.740 (0.723-0.759)
Fronto-pontine_tract_right	0.730 (0.717-0.745)	0.734 (0.725-0.758)
Fornix_left	1.519 (1.317-1.684)	1.436 (1.299-1.628)
Fornix_right	1.670 (1.469-1.899)	1.534 (1.334-1.886)
Inferior_cerebellar_peduncle_left	0.722 (0.665-0.745)	0.705 (0.669-0.809)
Inferior_cerebellar_peduncle_right	0.751 (0.687-0.851)	0.727 (0.702-0.815)
Inferior_occipito-frontal_fascicle_left	0.784 (0.761-0.801)	0.794 (0.780-0.824)
**Inferior_occipito-frontal_fascicle_right**	**0.787 (0.758-0.796)**	**0.801 (0.782-0.820)**
Inferior_longitudinal_fascicle_left	0.785 (0.765-0.812)	0.797 (0.771-0.815)
Inferior_longitudinal_fascicle_right	0.763 (0.744-0.775)	0.777 (0.753-0.811)
Middle_cerebellar_peduncle	0.745 (0.726-0.765)	0.744 (0.723-0.796)
Optic_radiation_left	0.790 (0.763-0.808)	0.788 (0.772-0.822)
Optic_radiation_right	0.786 (0.760-0.815)	0.798 (0.770-0.821)
**Parieto_occipital_pontine_left**	**0.748 (0.736-0.768)**	**0.761 (0.754-0.791)**
Parieto_occipital_pontine_right	0.747 (0.731-0.760)	0.756 (0.743-0.779)
Superior_cerebellar_peduncle_left	0.733 (0.723-0.764)	0.748 (0.718-0.773)
Superior_cerebellar_peduncle_right	0.729 (0.710-0.765)	0.752 (0.717-0.764)
**Superior_longitudinal_fascicle_I_left**	**0.706 (0.692-0.720)**	**0.725 (0.708-0.750)**
**Superior_longitudinal_fascicle_I_right**	**0.706 (0.697-0.726)**	**0.729 (0.709-0.748)**
**Superior_longitudinal_fascicle_II_left**	**0.704 (0.689-0.708)**	**0.710 (0.698-0.729)**
**Superior_longitudinal_fascicle_II_right**	**0.708 (0.702-0.729)**	**0.729 (0.712-0.749)**
**Superior_longitudinal_fascicle_III_left**	**0.703 (0.687-0.718)**	**0.715 (0.702-0.735)**
Superior_longitudinal_fascicle_III_right	0.711 (0.699-0.724)	0.715 (0.709-0.741)
Superior_Thalamic_Radiation_left	0.706 (0.689-0.715)	0.700 (0.692-0.720)
Superior_Thalamic_Radiation_right	0.696 (0.683-0.717)	0.695 (0.686-0.723)
Uncinate_fascicle_left	0.777 (0.763-0.790)	0.783 (0.765-0.796)
Uncinate_fascicle_right	0.767 (0.758-0.790)	0.786 (0.754-0.805)
**Corpus_Callosum-all**	**0.796 (0.768-0.809)**	**0.807 (0.790-0.834)**
Thalamo-prefrontal_left	0.754 (0.736-0.774)	0.759 (0.749-0.774)
Thalamo-prefrontal_right	0.754 (0.742-0.770)	0.764 (0.750-0.797)
Thalamo-premotor_left	0.722 (0.710-0.742)	0.723 (0.713-0.752)
Thalamo-premotor_right	0.715 (0.704-0.739)	0.719 (0.706-0.759)
Thalamo-precentral_left	0.711 (0.698-0.724)	0.716 (0.703-0.728)
Thalamo-precentral_right	0.708 (0.694-0.720)	0.708 (0.696-0.736)
Thalamo-postcentral_left	0.745 (0.729-0.758)	0.757 (0.734-0.774)
Thalamo-postcentral_right	0.730 (0.717-0.745)	0.739 (0.725-0.766)
Thalamo-parietal_left	0.773 (0.750-0.794)	0.790 (0.775-0.812)
Thalamo-parietal_right	0.772 (0.750-0.795)	0.783 (0.766-0.821)
Thalamo-occipital_left	0.795 (0.767-0.813)	0.792 (0.775-0.826)
Thalamo-occipital_right	0.789 (0.764-0.818)	0.803 (0.775-0.829)

Striato-fronto-orbital_left	0.771 (0.755-0.795)	0.779 (0.756-0.804)
Striato-fronto-orbital_right	0.757 (0.742-0.784)	0.769 (0.750-0.793)
**Striato-prefrontal_left**	**0.751 (0.737-0.761)**	**0.761 (0.751-0.777)**
**Striato-prefrontal_right**	**0.746 (0.737-0.759)**	**0.761 (0.744-0.780)**
Striato-premotor_left	0.709 (0.699-0.717)	0.718 (0.705-0.742)
Striato-premotor_right	0.707 (0.702-0.726)	0.720 (0.704-0.742)
Striato-precentral_left	0.708 (0.699-0.723)	0.719 (0.703-0.733)
Striato-precentral_right	0.702 (0.694-0.712)	0.710 (0.697-0.725)
Striato-postcentral_left	0.737 (0.725-0.757)	0.754 (0.735-0.771)
**Striato-postcentral_right**	**0.720 (0.708-0.738)**	**0.733 (0.724-0.758)**
**Striato-parietal_left**	**0.764 (0.748-0.782)**	**0.782 (0.775-0.806)**
**Striato-parietal_right**	**0.755 (0.739-0.769)**	**0.771 (0.750-0.795)**
Striato-occipital_left	0.777 (0.750-0.795)	0.783 (0.769-0.815)
Striato-occipital_right	0.769 (0.747-0.785)	0.781 (0.760-0.804)
Global WM metric value	0.778 (0.755-0.794)	0.779 (0.769-0.795)

**C. DTI - AD**		
	SRC athletes, median (IQR) *n*=20	Controls, median (IQR) *n*=21
**Arcuate_fascicle_left**	**1.035 (1.012-1.051)**	**1.044 (1.036-1.064)**
Arcuate_fascicle_right	1.035 (1.018-1.050)	1.044 (1.027-1.071)
Anterior_Thalamic_Radiation_left	1.067 (1.054-1.106)	1.073 (1.056-1.100)
Anterior_Thalamic_Radiation_right	1.067 (1.052-1.078)	1.072 (1.048-1.110)
Commissure_Anterior	1.196 (1.174-1.232)	1.212 (1.178-1.242)
Rostrum	1.275 (1.232-1.322)	1.294 (1.258-1.353)
Genu	1.143 (1.128-1.163)	1.152 (1.135-1.187)
Rostral_body_(Premotor)	1.176 (1.1699-1.190)	1.201 (1.163-1.222)
Anterior_midbody_(Primary_Motor)	1.165 (1.141-1.181)	1.181 (1.153-1.199)
Posterior_midbody_(Primary_Somatosensory)	1.183 (1.171-1.200)	1.193 (1.177-1.226)
Isthmus	1.161 (1.144-1.188)	1.175 (1.155-1.201)
Splenium	1.242 (1.192-1.275)	1.232 (1.196-1.266)
Cingulum_left	1.068 (1.056-1.084)	1.082 (1.062-1.099)
Cingulum_right	1.052 (1.040-1.065)	1.061 (1.047-1.076)
Corticospinal_tract_left	1.121 (1.112-1.134)	1.137 (1.116-1.149)
Corticospinal_tract_right	1.117 (1.109-1.133)	1.128 (1.106-1.147)
**Middle_longitudinal_fascicle_left**	**1.072 (1.060-1.086)**	**1.093 (1.080-1.109)**
**Middle_longitudinal_fascicle_right**	**1.084 (1.080-1.097)**	**1.102 (1.088-1.118)**
Fronto-pontine_tract_left	1.119 (1.099-1.134)	1.116 (1.106-1.150)
Fronto-pontine_tract_right	1.116 (1.091-1.121)	1.114 (1.105-1.151)
Fornix_left	2.189 (2.015-2.394)	2.069 (1.992-2.407)
Fornix_right	2.425 (2.113-2.752)	2.210 (2.026-2.708)
Inferior_cerebellar_peduncle_left	1.090 (0.995-1.193)	1.075 (1.013-1.205)
Inferior_cerebellar_peduncle_right	1.116 (1.023-1.286)	1.085 (1.027-1.127)
Inferior_occipito-frontal_fascicle_left	1.101 (1.083-1.121)	1.110 (1.095-1.147)
**Inferior_occipito-frontal_fascicle_right**	**1.084 (1.064-1.106)**	**1.108 (1.078-1.128)**
Inferior_longitudinal_fascicle_left	1.177 (1.127-1.227)	1.180 (1.147-1.221)
Inferior_longitudinal_fascicle_right	1.130 (1.101-1.165)	1.141 (1.103-1.164)

Middle_cerebellar_peduncle	1.161 (1.104-1.205)	1.161 (1.119-1.229)
Optic_radiation_left	1.163 (1-125-1.213)	1.156 (1.130-1.208)
Optic_radiation_right	1.160 (1.135-1.199)	1.167 (1.136-1.192)
**Parieto_occipital_pontine_left**	**1.129 (1.119-146)**	**1.143 (1.131-1.172)**
Parieto_occipital_pontine_right	1.138 (1.125-1.157)	1.150 (1.138-1.180)
Superior_cerebellar_peduncle_left	1.108 (1.067-1.147)	1.112 (1.084-1.169)
Superior_cerebellar_peduncle_right	1.082 (1.064-1.141)	1.126 (1.073-1.162)
Superior_longitudinal_fascicle_I_left	1.054 (1.041-1.063)	1.077 (1.046-1.098)
Superior_longitudinal_fascicle_I_right	1.053 (1.040-1.063)	1.070 (1.050-1.089)
Superior_longitudinal_fascicle_II_left	1.024 (1.016-1.036)	1.042 (1.018-1.059)
Superior_longitudinal_fascicle_II_right	1.026 (1.018-1.040)	1.045 (1.020-1.060)
Superior_longitudinal_fascicle_III_left	1.018 (1.003-1.043)	1.041 (1.020-1.057)
Superior_longitudinal_fascicle_III_right	1.019 (0.998-1.035)	1.018 (1.004-1.045)
Superior_Thalamic_Radiation_left	1.067 (1.052-1.079)	1.073 (1.050-1.094)
Superior_Thalamic_Radiation_right	1.065 (1.048-1.088)	1.083 (1.061-1.092)
Uncinate_fascicle_left	1.135 (1.105-1.150)	1.137 (1.113-1.160)
Uncinate_fascicle_right	1.103 (1.083-1.125)	1.120 (1.101-1.142)
**Corpus_Callosum-all**	**1.133 (1.120-1.149)**	**1.151 (1.130-1.177)**
Thalamo-prefrontal_left	1.066 (1.053-1.093)	1.079 (1.062-1.099)
Thalamo-prefrontal_right	1.059 (1.050-1.073)	1.078 (1.053-1.115)
Thalamo-premotor_left	1.047 (1.035-1.061)	1.061 (1.046-1.072)
Thalamo-premotor_right	1.038 (1.028-1.055)	1.045 (1.022-1.086)
Thalamo-precentral_left	1.056 (1.048-1.069)	1.072 (1.053-1.081)
Thalamo-precentral_right	1.049 (1.039-1.069)	1.062 (1.049-1.082)
Thalamo-postcentral_left	1.084 (1.070-1.099)	1.097 (1.075-1.120)
**Thalamo-postcentral_right**	**1.075 (1.064-1.110)**	**1.095 (1.083-1.125)**
**Thalamo-parietal_left**	**1.103 (1.093-1.136)**	**1.127 (1.113-1.159)**
Thalamo-parietal_right	1.122 (1.101-1.141)	1.136 (1.111-1.169)
Thalamo-occipital_left	1.159 (1.121-1.216)	1.155 (1.130-1.210)
Thalamo-occipital_right	1.154 (1.129-1.191)	1.162 (1.132-1.190)
Striato-fronto-orbital_left	1.098 (1.080-1.135)	1.104 (1.079-1.141)
Striato-fronto-orbital_right	1.105 (1.097-1.145)	1.125 (1.104-1.144)
**Striato-prefrontal_left**	**1.045 (1.040-1.070)**	**1.072 (1.048-1.083)**
**Striato-prefrontal_right**	**1.048 (1.038-1.062)**	**1.066 (1.046-1.081)**
**Striato-premotor_left**	**1.018 (1.001-1.023)**	**1.029 (1.014-1.049)**
Striato-premotor_right	1.020 (1.008-1.038)	1.038 (1.010-1.056)
Striato-precentral_left	1.040 (1.033-1.057)	1.062 (1.036-1.078)
Striato-precentral_right	1.038 (1.034-1.052)	1.054 (1.033-1.075)
Striato-postcentral_left	1.064 (1.054-1.085)	1.078 (1.065-1.108)
**Striato-postcentral_right**	**1.061 (1.043-1.082)**	**1.081 (1.064-1.111)**
**Striato-parietal_left**	**1.091 (1.081-1.117)**	**1.117 (1.100-1.141)**
**Striato-parietal_right**	**1.100 (1.081-1.121)**	**1.114 (1.099-1.140)**
Striato-occipital_left	1.150 (1.122-1.187)	1.148 (1.131-1.195)
Striato-occipital_right	1.123 (1.109-1.140)	1.135 (1.107-1.154)
Global WM metric value	1.135 (1.122-1.151)	1.140 (1.128-1.160)

**D. DTI - RD**	SRC athletes, median (IQR) *n*=20	Controls, median (IQR) *n*=21
Arcuate_fascicle_left	0.561 (0.545-0.577)	0.578 (0.553-0.588)
Arcuate_fascicle_right	0.574 (0.558-0.588)	0.585 (0.567-0.603)
Anterior_Thalamic_Radiation_left	0.627 (0.601-0.655)	0.626 (0.607-0.642)
Anterior_Thalamic_Radiation_right	0.631 (0.605-0.647)	0.624 (0.611-0.663)
Commissure_Anterior	0.671 (0.640-0.729)	0.697 (0.646-0.730)
Rostrum	0.581 (0.556-0.609)	0.596 (0.568-0.650)
Genu	0.636 (0.607-0.649)	0.643 (0.624-0.669)
Rostral_body_(Premotor)	0.599 (0.563-0.614)	0.604 (0.582-0.630)
Anterior_midbody_(Primary_Motor)	0.573 (0.532-0.585)	0.562 (0.545-0.590)
Posterior_midbody_(Primary_Somatosensory)	0.592 (0.553-0.604)	0.606 (0.569-0.621)
Isthmus	0.607 (0.574-0.630)	0.618 (0.602-0.656)
Splenium	0.613 (0.585-0.645)	0.644 (0.587-0.660)
**Cingulum_left**	**0.576 (0.558-0.585)**	**0.587 (0.577-0.615)**
**Cingulum_right**	**0.576 (0.566-0.594)**	**0.586 (0.573-0.612)**
Corticospinal_tract_left	0.495 (0.484-0.518)	0.497 (0.485-0.509)
Corticospinal_tract_right	0.491 (0.478-0.512)	0.489 (0.475-0.501)
**Middle_longitudinal_fascicle_left**	**0.576 (0.563-0.590)**	**0.601 (0.580-0.614)**
**Middle_longitudinal_fascicle_right**	**0.585 (0.562-0.592)**	**0.612 (0.586-0.625)**
Fronto-pontine_tract_left	0.563 (0.526-0.558)	0.549 (0.527-0.564)
Fronto-pontine_tract_right	0.536 (0.523-0.560)	0.545 (0.533-0.568)
Fornix_left	1.165 (1.002-1.272)	1.102 (0.989-1.239)
Fornix_right	1.291 (1.155-1.462)	1.196 (1.015-1.474)
Inferior_cerebellar_peduncle_left	0.529 (0.501-0.588)	0.524 (0.488-0.570)
Inferior_cerebellar_peduncle_right	0.542 (0.524-0.621)	0.552 (0.533-0.588)
**Inferior_occipito-frontal_fascicle_left**	**0.625 (0.599-0.636)**	**0.635 (0.622-0.664)**
**Inferior_occipito-frontal_fascicle_right**	**0.635 (0.603-0.649)**	**0.647 (0.630-0.669)**
Inferior_longitudinal_fascicle_left	0.597 (0.573-0.612)	0.602 (0.580-0.621)
Inferior_longitudinal_fascicle_right	0.574 (0.544-0.609)	0.587 (0.575-0.624)
Middle_cerebellar_peduncle	0.537 (0.532-0.562)	0.541 (0.518-0.565)
Optic_radiation_left	0.601 (0.574-0.621)	0.616 (0.587-0.631)
Optic_radiation_right	0.596 (0.566-0.630)	0.613 (0.585-0.636)
Parieto_occipital_pontine_left	0.557 (0.542-0.581)	0.569 (0.558-0.603)
Parieto_occipital_pontine_right	0.552 (0.531-0.562)	0.557 (0.547-0.581)
Superior_cerebellar_peduncle_left	0.555 (0.544-0.575)	0.561 (0.531-0.575)
Superior_cerebellar_peduncle_right	0.547 (0.534-0.574)	0.559 (0.536-0.574)
Superior_longitudinal_fascicle_I_left	0.527 (0.513-0.550)	0.542 (0.525-0.584)
**Superior_longitudinal_fascicle_I_right**	**0.536 (0.528-0.554)**	**0.553 (0.539-0.579)**
Superior_longitudinal_fascicle_II_left	0.538 (0.525-0.546)	0.552 (0.530-0.571)
**Superior_longitudinal_fascicle_II_right**	**0.552 (0.540-0.571)**	**0.574 (0.557-0.598)**
**Superior_longitudinal_fascicle_III_left**	**0.543 (0.528-0.548)**	**0.566 (0.537-0.580)**
Superior_longitudinal_fascicle_III_right	0.561 (0.545-0.573)	0.571 (0.554-0.590)
Superior_Thalamic_Radiation_left	0.522 (0.502-0.534)	0.520 (0.500-0.539)
Superior_Thalamic_Radiation_right	0.511 (0.500-0.528)	0.509 (0.493-0.534)
Uncinate_fascicle_left	0.599 (0.578-0.619)	0.608 (0.587-0.618)

Uncinate_fascicle_right	0.600 (0.587-0.672)	0.617 (0.588-0.639)
Corpus_Callosum-all	0.628 (0.592-0.642)	0.637 (0.617-0.669)
Thalamo-prefrontal_left	0.596 (0.574-0.614)	0.605 (0.587-0.619)
Thalamo-prefrontal_right	0.603 (0.586-0.624)	0.612 (0.592-0.644)
Thalamo-premotor_left	0.563 (0.545-0.581)	0.561 (0.549-0.595)
Thalamo-premotor_right	0.558 (0.539-0.584)	0.563 (0.547-0.593)
Thalamo-precentral_left	0.540 (0.519-0.551)	0.539 (0.527-0.559)
Thalamo-precentral_right	0.535 (0.518-0.552)	0.529 (0.523-0.570)
Thalamo-postcentral_left	0.568 (0.553-0.591)	0.580 (0.558-0.610)
Thalamo-postcentral_right	0.554 (0.538-0.575)	0.573 (0.539-0.591)
Thalamo-parietal_left	0.607 (0.574-0.624)	0.620 (0.595-0.645)
Thalamo-parietal_right	0.599 (0.570-0.624)	0.607 (0.589-0.649)
Thalamo-occipital_left	0.608 (0.581-0.628)	0.620 (0.592-0.636)
Thalamo-occipital_right	0.603 (0.575-0.641)	0.624 (0.597-0.648)
Striato-fronto-orbital_left	0.606 (0.587-0.626)	0.605 (0.593-0.641)
Striato-fronto-orbital_right	0.578 (0.560-0.612)	0.589 (0.569-0.626)
Striato-prefrontal_left	0.600 (0.584-0.612)	0.618 (0.594-0.633)
Striato-prefrontal_right	0.596 (0.586-0.612)	0.606 (0.588-0.634)
Striato-premotor_left	0.555 (0.543-0.567)	0.564 (0.544-0.590)
Striato-premotor_right	0.551 (0.539-0.572)	0.566 (0.547-0.578)
Striato-precentral_left	0.542 (0.529-0.552)	0.549 (0.531-0.573)
Striato-precentral_right	0.530 (0.523-0.550)	0.539 (0.523-0.556)
Striato-postcentral_left	0.571 (0.557-0.589)	0.588 (0.570-0.614)
Striato-postcentral_right	0.548 (0.536-0.568)	0.568 (0.543-0.584)
**Striato-parietal_left**	**0.593 (0.577-0.619)**	**0.612 (0.597-0.641)**
**Striato-parietal_right**	**0.585 (0.561-0.598)**	**0.601 (0.581-0.622)**
Striato-occipital_left	0.592 (0.566-0.600)	0.600 (0.587-0.628)
**Striato-occipital_right**	**0.589 (0.565-0.617)**	**0.606 (0.588-0.630)**
Global WM metric value	0.599 (0.572-0.610)	0.600 (0.585-0.619)

**E. DKI - FA**		
	SRC athletes, median (IQR) *n*=19	Controls, median (IQR) *n*=20
**Arcuate_fascicle_left**	**0.427 (0.418-0.445)**	**0.403 (0.389-0.418)**
**Arcuate_fascicle_right**	**0.408 (0.395-0.423)**	**0.399 (0.386-0.412)**
**Anterior_Thalamic_Radiation_left**	**0.417 (0.408-0.439)**	**0.399 (0.391-0.414)**
**Anterior_Thalamic_Radiation_right**	**0.402 (0.391-0.422)**	**0.394 (0.383-0.401)**
Commissure_Anterior	0.419 (0.401-0.426)	0.391 (0.361-0.437)
**Rostrum**	**0.520 (0.480-0.548)**	**0.480 (0.463-0.503)**
**Genu**	**0.421 (0.403-0.435)**	**0.396 (0.386-0.403)**
**Rostral_body_(Premotor)**	**0.437 (0.427-0.452)**	**0.421 (0.415-0.439)**
Anterior_midbody_(Primary_Motor)	0.474 (0.458-0.481)	0.460 (0.449-0.469)
Posterior_midbody_(Primary_Somatosensory)	0.461 (0.454-0.479)	0.454 (0.444-0.468)
**Isthmus**	**0.459 (0.452-0.469)**	**0.444 (0.429-0.454)**
**Splenium**	**0.466 (0.446-0.492)**	**0.444 (0.430-0.469)**
**Cingulum_left**	**0.447 (0.432-0.451)**	**0.418 (0.403-0.433)**
**Cingulum_right**	**0.421 (0.402-0.429)**	**0.393 (0.383-0.396)**

Corticospinal_tract_left	0.531 (0.521-0.541)	0.522 (0.507-0.535)
Corticospinal_tract_right	0.522 (0.509-0.527)	0.512 (0.504-0.523)
**Middle_longitudinal_fascicle_left**	**0.449 (0.442-0.460)**	**0.421 (0.409-0.436)**
**Middle_longitudinal_fascicle_right**	**0.444 (0.438-0.463)**	**0.432 (0.414-0.445)**
**Fronto-pontine_tract_left**	**0.496 (0.475-0.508)**	**0.480 (0.474-0.490)**
**Fronto-pontine_tract_right**	**0.478 (0.463-0.488)**	**0.465 (0.456-0.479)**
Fornix_left	0.505 (0.490-0.512)	0.497 (0.465-0.514)
Fornix_right	0.495 (0.470-0.514)	0.495 (0.471-0.525)
Inferior_cerebellar_peduncle_left	0.533 (0.518-0.556)	0.518 (0.504-0.542)
Inferior_cerebellar_peduncle_right	0.502 (0.488-0.538)	0.497 (0.485-0.517)
**Inferior_occipito-frontal_fascicle_left**	**0.429 (0.415-0.442)**	**0.405 (0.395-0.422)**
**Inferior_occipito-frontal_fascicle_right**	**0.408 (0.395-0.428)**	**0.390 (0.380-0.401)**
Inferior_longitudinal_fascicle_left	0.473 (0.449-0.491)	0.454 (0.433-0.467)
**Inferior_longitudinal_fascicle_right**	**0.449 (0.437-0.500)**	**0.434 (0.418-0.459)**
Middle_cerebellar_peduncle	0.552 (0.529-0.573)	0.543 (0.523-0.555)
**Optic_radiation_left**	**0.467 (0.459-0.491)**	**0.446 (0.435-0.462)**
**Optic_radiation_right**	**0.471 (0.452-0.481)**	**0.449 (0.440-0.459)**
**Parieto_occipital_pontine_left**	**0.491 (0.483-0.497)**	**0.476 (0.470-0.482)**
**Parieto_occipital_pontine_right**	**0.493 (0.480-0.498)**	**0.480 (0.472-0.491)**
Superior_cerebellar_peduncle_left	0.522 (0.506-0.536)	0.511 (0.506-0.524)
Superior_cerebellar_peduncle_right	0.519 (0.506-0.534)	0.515 (0.502-0.527)
**Superior_longitudinal_fascicle_I_left**	**0.461 (0.451-0.472)**	**0.446 (0.434-0.457)**
**Superior_longitudinal_fascicle_I_right**	**0.444 (0.432-0.453)**	**0.427 (0.417-0.433)**
**Superior_longitudinal_fascicle_II_left**	**0.443 (0.430-0.448)**	**0.422 (0.411-0.435)**
**Superior_longitudinal_fascicle_II_right**	**0.427 (0.414-0.432)**	**0.404 (0.402-0.419)**
**Superior_longitudinal_fascicle_III_left**	**0.433 (0.426-0.455)**	**0.411 (0.401-0.428)**
Superior_longitudinal_fascicle_III_right	0.408 (0.393-0.421)	0.397 (0.386-0.411)
Superior_Thalamic_Radiation_left	0.504 (0.491-0.516)	0.496 (0.479-0.513)
Superior_Thalamic_Radiation_right	0.481 (0.471-0.499)	0.477 (0.464-0.493)
**Uncinate_fascicle_left**	**0.444 (0.413-0.452)**	**0.421 (0.400-0.434)**
**Uncinate_fascicle_right**	**0.402 (0.387-0.435)**	**0.384 (0.367-0.413)**
**Corpus_Callosum-all**	**0.432 (0.419-0.443)**	**0.410 (0.402-0.420)**
**Thalamo-prefrontal_left**	**0.431 (0.425-0.448)**	**0.415 (0.406-0.421)**
**Thalamo-prefrontal_right**	**0.409 (0.398-0.421)**	**0.390 (0.384-0.399)**
**Thalamo-premotor_left**	**0.440 (0.429-0.450)**	**0.431 (0.420-0.437)**
**Thalamo-premotor_right**	**0.437 (0.423-0.445)**	**0.421 (0.416-0.428)**
**Thalamo-precentral_left**	**0.468 (0.464-0.480)**	**0.459 (0.449-0.465)**
Thalamo-precentral_right	0.452 (0.443-0.466)	0.444 (0.432-0.459)
**Thalamo-postcentral_left**	**0.455 (0.445-0.471)**	**0.443 (0.432-0.463)**
Thalamo-postcentral_right	0.455 (0.439-0.465)	0.444 (0.432-0.458)
**Thalamo-parietal_left**	**0.450 (0.443-0.463)**	**0.435 (0.431-0.442)**
**Thalamo-parietal_right**	**0.451 (0.445-0.466)**	**0.442 (0.427-0.453)**
**Thalamo-occipital_left**	**0.461 (0.454-0.487)**	**0.443 (0.430-0.456)**
**Thalamo-occipital_right**	**0.463 (0.442-0.477)**	**0.440 (0.430-0.452)**
**Striato-fronto-orbital_left**	**0.450 (0.426-0.459)**	**0.420 (0.413-0.439)**
**Striato-fronto-orbital_right**	**0.443 (0.431-0.483)**	**0.415 (0.405-0.439)**
**Striato-prefrontal_left**	**0.421 (0.411-0.439)**	**0.399 (0.392-0.409)**

**Striato-prefrontal_right**	**0.408 (0.390-0.418)**	**0.384 (0.379-0.394)**
**Striato-premotor_left**	**0.418 (0.411-0.429)**	**0.404 (0.394-0.420)**
**Striato-premotor_right**	**0.419 (0.402-0.428)**	**0.402 (0.395-0.413)**
**Striato-precentral_left**	**0.456 (0.449-0.467)**	**0.442 (0.431-0.450)**
Striato-precentral_right	0.450 (0.434-0.459)	0.435 (0.428-0.448)
**Striato-postcentral_left**	**0.439 (0.437-0.450)**	**0.430 (0.418-0.443)**
Striato-postcentral_right	0.452 (0.430-0.460)	0.433 (0.426-0.453)
**Striato-parietal_left**	**0.448 (0.439-0.455)**	**0.429 (0.421-0.433)**
**Striato-parietal_right**	**0.454 (0.441-0.462)**	**0.439 (0.425-0.446)**
**Striato-occipital_left**	**0.464 (0.455-0.494)**	**0.447 (0.433-0.450)**
**Striato-occipital_right**	**0.458 (0.434-0.479)**	**0.434 (0.421-0.447)**
**Global WM metric value**	**0.459 (0.448-0.464)**	**0.438 (0.436-0.449)**

F. DKI – MD

	SRC athletes, median (IQR) *n*=19	Controls, median (IQR) *n*=20
Arcuate_fascicle_left	0.841 (0.826-0.878)	0.861 (0.849-0.880)
Arcuate_fascicle_right	0.887 (0.876-0.914)	0.897 (0.883-0.920)
Anterior_Thalamic_Radiation_left	0.908 (0.894-0.926)	0.909 (0.892-0.943)
Anterior_Thalamic_Radiation_right	0.945 (0.920-0.965)	0.936 (0.917-0.976)
Commissure_Anterior	1.039 (0.999-1.109)	1.051 (1.014-1.098)
Rostrum	1.014 (0.994-1.092)	1.067 (1.011-1.101)
Genu	0.987 (0.963-1.001)	0.999 (0.977-1.020)
Rostral_body_(Premotor)	0.953 (0.925-0.983)	0.971 (0.947-1.006)
Anterior_midbody_(Primary_Motor)	0.914 (0.890-0.946)	0.929 (0.918-0.942)
Posterior_midbody_(Primary_Somatosensory)	0.957 (0.920-0.997)	0.974 (0.955-1.002)
**Isthmus**	**0.963 (0.938-0.993)**	**0.982 (0.970-1.009)**
Splenium	1.021 (0.963-1.057)	1.025 (0.979-1.073)
**Cingulum_left**	**0.891 (0.881-0.911)**	**0.916 (0.903-0.930)**
**Cingulum_right**	**0.895 (0.870-0.907)**	**0.911 (0.898-0.936)**
Corticospinal_tract_left	0.851 (0.835-0.873)	0.865 (0.856-0.877)
Corticospinal_tract_right	0.869 (0.855-0.886)	0.879 (0.859-0.893)
**Middle_longitudinal_fascicle_left**	**0.886 (0.872-0.900)**	**0.915 (0.891-0.927)**
**Middle_longitudinal_fascicle_right**	**0.911 (0.895-0.930)**	**0.941 (0.924-0.951)**
**Fronto-pontine_tract_left**	**0.896 (0.884-0.907)**	**0.915 (0.896-0.928)**
**Fronto-pontine_tract_right**	**0.906 (0.891-0.927)**	**0.924 (0.903-0.947)**
Fornix_left	1.879 (1.657-2.137)	1.826 (1.689-2.019)
Fornix_right	1.875 (1.706-2.234)	1.945 (1.836-2.124)
Inferior_cerebellar_peduncle_left	0.825 (0.798-0.864)	0.842 (0.826-0.901)
Inferior_cerebellar_peduncle_right	0.886 (0.843-0.919)	0.879 (0.853-0.934)
Inferior_occipito-frontal_fascicle_left	0.964 (0.932-0.978)	0.974 (0.943-0.996)
Inferior_occipito-frontal_fascicle_right	0.971 (0.922-0.989)	0.984 (0.957-1.022)
Inferior_longitudinal_fascicle_left	0.936 (0.906-0.956)	0.939 (0.911-0.954)
Inferior_longitudinal_fascicle_right	0.926 (0.904-0.946)	0.941 (0.915-0.968)
Middle_cerebellar_peduncle	0.898 (0.857-0.924)	0.906 (0.891-0.925)
Optic_radiation_left	0.947 (0.912-0.978)	0.955 (0.921-0.991)
Optic_radiation_right	0.958 (0.924-0.982)	0.965 (0.927-0.995)

**Parieto_occipital_pontine_left**	**0.922 (0.899-0.946)**	**0.938 (0.923-0.972)**
**Parieto_occipital_pontine_right**	**0.930 (0.911-0.954)**	**0.952 (0.942-0.977)**
Superior_cerebellar_peduncle_left	0.908 (0.888-0.933)	0.922 (0.895-0.967)
Superior_cerebellar_peduncle_right	0.925 (0.897-0.949)	0.932 (0.908-0.959)
**Superior_longitudinal_fascicle_I_left**	**0.857 (0.829-0.880)**	**0.881 (0.854-0.919)**
**Superior_longitudinal_fascicle_I_right**	**0.871 (0.859-0.896)**	**0.895 (0.880-0.924)**
**Superior_longitudinal_fascicle_II_left**	**0.843 (0.824-0.866)**	**0.858 (0.845-0.892)**
**Superior_longitudinal_fascicle_II_right**	**0.862 (0.850-0.886)**	**0.889 (0.871-0.919)**
**Superior_longitudinal_fascicle_III_left**	**0.836 (0.809-0.866)**	**0.862 (0.843-0.880)**
Superior_longitudinal_fascicle_III_right	0.871 (0.846-0.894)	0.868 (0.859-0.902)
Superior_Thalamic_Radiation_left	0.814 (0.799-0.834)	0.825 (0.809-0.840)
Superior_Thalamic_Radiation_right	0.825 (0.811-0.845)	0.833 (0.814-0.846)
Uncinate_fascicle_left	0.914 (0.887-0.934)	0.928 (0.908-0.949)
Uncinate_fascicle_right	0.930 (0.922-0.961)	0.941 (0.921-0.968)
Corpus_Callosum-all	0.966 (0.950-0.984)	0.984 (0.963-1.005)
Thalamo-prefrontal_left	0.888 (0.883-0.907)	0.905 (0.894-0.924)
Thalamo-prefrontal_right	0.919 (0.901-0.940)	0.926 (0.912-0.954)
Thalamo-premotor_left	0.832 (0.820-0.856)	0.851 (0.835-0.866)
Thalamo-premotor_right	0.851 (0.837-0.879)	0.865 (0.846-0.888)
**Thalamo-precentral_left**	**0.828 (0.810-0.846)**	**0.851 (0.837-0.867)**
Thalamo-precentral_right	0.855 (0.839-0.874)	0.865 (0.851-0.877)
Thalamo-postcentral_left	0.877 (0.860-0.926)	0.899 (0.893-0.943)
Thalamo-postcentral_right	0.907 (0.871-0.938)	0.925 (0.898-0.957)
**Thalamo-parietal_left**	**0.930 (0.898-0.954)**	**0.950 (0.931-0.984)**
**Thalamo-parietal_right**	**0.943 (0.908-0.978)**	**0.970 (0.945-0.987)**
Thalamo-occipital_left	0.950 (0.916-0.983)	0.959 (0.926-0.995)
Thalamo-occipital_right	0.963 (0.927-0.992)	0.973 (0.934-1.006)
Striato-fronto-orbital_left	0.926 (0.905-0.949)	0.934 (0.912-0.945)
Striato-fronto-orbital_right	0.943 (0.904-0.981)	0.939 (0.913-0.978)
Striato-prefrontal_left	0.902 (0.884-0.918)	0.915 (0.902-0.946)
Striato-prefrontal_right	0.921 (0.899-0.940)	0.921 (0.914-0.952)
Striato-premotor_left	0.834 (0.814-0.854)	0.852 (0.835-0.873)
Striato-premotor_right	0.854 (0.833-0.873)	0.870 (0.843-0.885)
Striato-precentral_left	0.824 (0.817-0.858)	0.858 (0.841-0.874)
Striato-precentral_right	0.856 (0.835-0.868)	0.861 (0.849-0.882)
Striato-postcentral_left	0.877 (0.855-0.913)	0.897 (0.890-0.937)
Striato-postcentral_right	0.895 (0.873-0.921)	0.907 (0.894-0.947)
**Striato-parietal_left**	**0.923 (0.894-0.947)**	**0.946 (0.930-0.978)**
**Striato-parietal_right**	**0.926 (0.904-0.947)**	**0.951 (0.937-0.973)**
Striato-occipital_left	0.950 (0.917-0.976)	0.952 (0.920-1.006)
Striato-occipital_right	0.944 (0.910-0.969)	0.962 (0.934-0.991)
Global WM metric value	0.940 (0.918-0.957)	0.948 (0.935-0.959)

**G. DKI - AD**		
	SRC athletes, median (IQR) *n*=19	Controls, median (IQR) *n*=20
Arcuate_fascicle_left	1.263 (1.221-1.280)	1.252 (1.235-1.273)

Arcuate_fascicle_right	1.294 (1.264-1.320)	1.289 (1.276-1.320)
Anterior_Thalamic_Radiation_left	1.323 (1.302-1.355)	1.306 (1.285-1.334)
Anterior_Thalamic_Radiation_right	1.368 (1.326-1.379)	1.342 (1.318-1.381)
Commissure_Anterior	1.475 (1.463-1.575)	1.498 (1.472-1.525)
Rostrum	1.610 (1.587-1.720)	1.648 (1.571-1.722)
Genu	1.438 (1.421-1.460)	1.434 (1.407-1.479)
Rostral_body_(Premotor)	1.437 (1.388-1.474)	1.461 (1.412-1.480)
Anterior_midbody_(Primary_Motor)	1.425 (1.388-1.449)	1.429 (1.400-1.458)
Posterior_midbody_(Primary_Somatosensory)	1.477 (1.412-1.521)	1.468 (1.453-1.528)
Isthmus	1.475 (1.434-1.500)	1.474 (1.446-1.500)
Splenium	1.583 (1.517-1.615)	1.528 (1.485-1.607)
Cingulum_left	1.330 (1.318-1.358)	1.340 (1.316-1.362)
Cingulum_right	1.308 (1.276-1.332)	1.306 (1.278-1.334)
Corticospinal_tract_left	1.394 (1.374-1.437)	1.411 (1.384-1.427)
Corticospinal_tract_right	1.409 (1.397-1.428)	1.417 (1.402-1.442)
Middle_longitudinal_fascicle_left	1.332 (1.307-1.348)	1.336 (1.314-1.364)
Middle_longitudinal_fascicle_right	1.371 (1.334-1.410)	1.380 (1.363-1.405)
Fronto-pontine_tract_left	1.426 (1.370-1.446)	1.426 (1.390-1.443)
Fronto-pontine_tract_right	1.417 (1.369-1.440)	1.430 (1.393-1.456)
Fornix_left	2.863 (2.657-3.470)	2.879 (2.705-3.190)
Fornix_right	3.020 (2.791-3.506)	3.023 (2.913-3.410)
Inferior_cerebellar_peduncle_left	1.361 (1.307-1.434)	1.379 (1.332-1.454)
Inferior_cerebellar_peduncle_right	1.416 (1.346-1.489)	1.401 (1.331-1.471)
Inferior_occipito-frontal_fascicle_left	1.408 (1.372-1.449)	1.411 (1.362-1.443)
Inferior_occipito-frontal_fascicle_right	1.408 (1.359-1.434)	1.410 (1.367-1.442)
Inferior_longitudinal_fascicle_left	1.447 (1.360-1.487)	1.410 (1.394-1.444)
Inferior_longitudinal_fascicle_right	1.402 (1.354-1.438)	1.385 (1.364-1.444)
Middle_cerebellar_peduncle	1.480 (1.426-1.541)	1.483 (1.460-1.498)
Optic_radiation_left	1.453 (1.396-1.506)	1.434 (1.395-1.465)
Optic_radiation_right	1.457 (1.392-1.480)	1.442 (1.387-1.484)
Parieto_occipital_pontine_left	1.430 (1.400-1.465)	1.442 (1.422-1.465)
Parieto_occipital_pontine_right	1.456 (1.418-1.481)	1.470 (1.457-1.499)
Superior_cerebellar_peduncle_left	1.485 (1.447-1.509)	1.502 (1.448-1.589)
Superior_cerebellar_peduncle_right	1.520 (1.435-1.559)	1.511 (1.444-1.579)
Superior_longitudinal_fascicle_I_left	1.294 (1.259-1.322)	1.314 (1.286-1.352)
Superior_longitudinal_fascicle_I_right	1.296 (1.273-1.338)	1.321 (1.301-1.343)
Superior_longitudinal_fascicle_II_left	1.265 (1.235-1.289)	1.264 (1.242-1.294)
Superior_longitudinal_fascicle_II_right	1.277 (1.261-1.308)	1.289 (1.272-1.324)
Superior_longitudinal_fascicle_III_left	1.238 (1.207-1.276)	1.260 (1.236-1.274)
Superior_longitudinal_fascicle_III_right	1.249 (1.226-1.281)	1.250 (1.238-1.281)
Superior_Thalamic_Radiation_left	1.294 (1.282-1.313)	1.305 (1.252-1.323)
Superior_Thalamic_Radiation_right	1.286 (1.273-1.303)	1.308 (1.285-1.325)
Uncinate_fascicle_left	1.369 (1.335-1.412)	1.363 (1.340-1.383)
Uncinate_fascicle_right	1.362 (1.328-1.406)	1.353 (1.327-1.371)
Corpus_Callosum-all	1.432 (1.407-1.452)	1.427 (1.400-1.459)
Thalamo-prefrontal_left	1.316 (1.295-1.349)	1.316 (1.297-1.336)
Thalamo-prefrontal_right	1.341 (1.306-1.353)	1.322 (1.298-1.345)

Thalamo-premotor_left	1.255 (1.235-1.268)	1.261 (1.252-1.280)
Thalamo-premotor_right	1.271 (1.249-1.287)	1.270 (1.254-1.295)
Thalamo-precentral_left	1.269 (1.259-1.301)	1.296 (1.262-1.308)
Thalamo-precentral_right	1.303 (1.276-1.319)	1.301 (1.279-1.319)
Thalamo-postcentral_left	1.350 (1.307-1.375)	1.341 (1.314-1.375)
Thalamo-postcentral_right	1.368 (1.301-1.403)	1.377 (1.350-1.437)
Thalamo-parietal_left	1.394 (1.347-1.426)	1.396 (1.386-1.416)
Thalamo-parietal_right	1.418 (1.363-1.459)	1.428 (1.401-1.466)
Thalamo-occipital_left	1.453 (1.395-1.509)	1.438 (1.390-1.464)
Thalamo-occipital_right	1.461 (1.388-1.474)	1.443 (1.381-1.483)
Striato-fronto-orbital_left	1.366 (1.356-1.398)	1.374 (1.331-1.385)
Striato-fronto-orbital_right	1.408 (1.375-1.467)	1.396 (1.369-1.439)
Striato-prefrontal_left	1.321 (1.303-1.345)	1.312 (1.293-1.343)
Striato-prefrontal_right	1.332 (1.302-1.347)	1.311 (1.293-1.348)
Striato-premotor_left	1.225 (1.199-1.245)	1.235 (1.205-1.246)
Striato-premotor_right	1.247 (1.228-1.279)	1.253 (1.232-1.276)
Striato-precentral_left	1.256 (1.244-1.296)	1.281 (1.243-1.296)
Striato-precentral_right	1.283 (1.266-1.317)	1.293 (1.265-1.309)
Striato-postcentral_left	1.332 (1-284-1.342)	1.313 (1.291-1.361)
Striato-postcentral_right	1.342 (1.293-1.376)	1.347 (1.327-1.391)
Striato-parietal_left	1.384 (1.335-1.400)	1.387 (1.364-1.415)
Striato-parietal_right	1.390 (1.358-1.427)	1.403 (1.385-1.425)
Striato-occipital_left	1.452 (1.402-1.498)	1.429 (1.399-1.480)
Striato-occipital_right	1.431 (1.377-1.454)	1.427 (1.374-1.458)
Global WM metric value	1.436 (1.394-1.448)	1.413 (1.401-1.447)

**H. DKI - RD**		
	SRC athletes, median (IQR) *n*=19	Controls, median (IQR) *n*=20
**Arcuate_fascicle_left**	**0.649 (0.625-0.675)**	**0.674 (0.651-0.687)**
Arcuate_fascicle_right	0.689 (0.676-0.718)	0.698 (0.687-0.727)
Anterior_Thalamic_Radiation_left	0.700 (0.686-0.719)	0.713 (0.691-0.741)
Anterior_Thalamic_Radiation_right	0.741 (0.702-0.760)	0.736 (0.713-0.784)
Commissure_Anterior	0.795 (0.770-0.862)	0.826 (0.777-0.886)
**Rostrum**	**0.710 (0.684-0.745)**	**0.771 (0.716-0.804)**
**Genu**	**0.759 (0.733-0.780)**	**0.785 (0.754-0.802)**
Rostral_body_(Premotor)	0.718 (0.690-0.754)	0.729 (0.709-0.776)
Anterior_midbody_(Primary_Motor)	0.699 (0.641-0.695)	0.680 (0.671-0.701)
Posterior_midbody_(Primary_Somatosensory)	0.711 (0.667-0.739)	0.727 (0.702-0.756)
**Isthmus**	**0.710 (0.686-0.743)**	**0.740 (0.724-0.763)**
Splenium	0.744 (0.711-0.779)	0.771 (0.726-0.800)
**Cingulum_left**	**0.671 (0.664-0.702)**	**0.705 (0.690-0.726)**
**Cingulum_right**	**0.689 (0.675-0.700)**	**0.717 (0.702-0.740)**
Corticospinal_tract_left	0.577 (0.560-0.597)	0.592 (0.580-0.610)
Corticospinal_tract_right	0.587 (0.587-0.618)	0.611 (0.587-0.623)
**Middle_longitudinal_fascicle_left**	**0.665 (0.647-0.682)**	**0.706 (0.680-0.718)**
**Middle_longitudinal_fascicle_right**	**0.685 (0.667-0.703)**	**0.726 (0.699-0.733)**

**Fronto-pontine_tract_left**	**0.635 (0.618-0.657)**	**0.662 (0.643-0.675)**
**Fronto-pontine_tract_right**	**0.654 (0.632-0.671)**	**0.682 (0.646-0.691)**
Fornix_left	1.383 (1.158-1.470)	1.291 (1.169-1.423)
Fornix_right	1.353 (1.234-1.509)	1.393 (1.275-1.506)
**Inferior_cerebellar_peduncle_left**	**0.568 (0.548-0.588)**	**0.596 (0.565-0.633)**
Inferior_cerebellar_peduncle_right	0.614 (0.587-0.662)	0.629 (0.615-0.661)
Inferior_occipito-frontal_fascicle_left	0.740 (0.712-0.748)	0.756 (0.727-0.784)
**Inferior_occipito-frontal_fascicle_right**	**0.754 (0.714-0.776)**	**0.778 (0.757-0.818)**
Inferior_longitudinal_fascicle_left	0.680 (0.662-0.709)	0.695 (0.670-0.723)
Inferior_longitudinal_fascicle_right	0.689 (0.656-0.713)	0.703 (0.682-0.729)
Middle_cerebellar_peduncle	0.604 (0.579-0.634)	0.616 (0.604-0.640)
Optic_radiation_left	0.697 (0.676-0.724)	0.712 (0.686-0.755)
Optic_radiation_right	0.707 (0.668-0.728)	0.724 (0.695-0.748)
**Parieto_occipital_pontine_left**	**0.662 (0.649-0.689)**	**0.691 (0.673-0.716)**
**Parieto_occipital_pontine_right**	**0.671 (0.657-0.691)**	**0.695 (0.680-0.717)**
**Superior_cerebellar_peduncle_left**	**0.624 (0.607-0.633)**	**0.638 (0.624-0.659)**
Superior_cerebellar_peduncle_right	0.629 (0.618-0.643)	0.642 (0.629-0.669)
**Superior_longitudinal_fascicle_I_left**	**0.634 (0.615-0.666)**	**0.662 (0.640-0.699)**
**Superior_longitudinal_fascicle_I_right**	**0.655 (0.645-0.679)**	**0.687 (0.669-0.713)**
**Superior_longitudinal_fascicle_II_left**	**0.632 (0.618-0.658)**	**0.657 (0.649-0.692)**
**Superior_longitudinal_fascicle_II_right**	**0.662 (0.652-0.689)**	**0.690 (0.675-0.717)**
**Superior_longitudinal_fascicle_III_left**	**0.639 (0.612-0.669)**	**0.671 (0.650-0.688)**
Superior_longitudinal_fascicle_III_right	0.680 (0.648-0.696)	0.690 (0.664-0.714)
Superior_Thalamic_Radiation_left	0.534 (0.553-0.596)	0.581 (0.568-0.601)
Superior_Thalamic_Radiation_right	0.594 (0.573-0.614)	0.598 (0.586-0.618)
**Uncinate_fascicle_left**	**0.680 (0.649-0.714)**	**0.704 (0.691-0.740)**
Uncinate_fascicle_right	0.723 (0.680-0.741)	0.741 (0.705-0.777)
**Corpus_Callosum-all**	**0.730 (0.713-0.759)**	**0.761 (0.740-0.773)**
**Thalamo-prefrontal_left**	**0.674 (0.658-0.695)**	**0.700 (0.687-0.722)**
Thalamo-prefrontal_right	0.716 (0.699-0.738)	0.730 (0.710-0.759)
Thalamo-premotor_left	0.627 (0.611-0.652)	0.642 (0.632-0.663)
Thalamo-premotor_right	0.643 (0.633-0.675)	0.661 (0.645-0.685)
**Thalamo-precentral_left**	**0.609 (0.586-0.621)**	**0.625 (0.614-0.644)**
Thalamo-precentral_right	0.636 (0.618-0.654)	0.645 (0.633-0.666)
Thalamo-postcentral_left	0.651 (0.637-0.697)	0.693 (0.661-0.729)
Thalamo-postcentral_right	0.675 (0.663-0.709)	0.704 (0.675-0.735)
**Thalamo-parietal_left**	**0.691 (0.673-0.725)**	**0.728 (0.713-0.755)**
**Thalamo-parietal_right**	**0.708 (0.685-0.736)**	**0.743 (0.714-0.756)**
Thalamo-occipital_left	0.704 (0.685-0.732)	0.718 (0.694-0.764)
Thalamo-occipital_right	0.714 (0.677-0.739)	0.737 (0.707-0.761)
**Striato-fronto-orbital_left**	**0.697 (0.671-0.720)**	**0.720 (0.691-0.728)**
Striato-fronto-orbital_right	0.711 (0.662-0.733)	0.727 (0.695-0.760)
**Striato-prefrontal_left**	**0.692 (0.675-0.714)**	**0.721 (0.703-0.742)**
**Striato-prefrontal_right**	**0.711 (0.698-0.738)**	**0.732 (0.718-0.762)**
**Striato-premotor_left**	**0.635 (0.624-0.667)**	**0.660 (0.646-0.683)**
Striato-premotor_right	0.661 (0.633-0.677)	0.673 (0.658-0.687)
**Striato-precentral_left**	**0.611 (0.599-0.643)**	**0.643 (0.628-0.658)**

Striato-precentral_right	0.642 (0.622-0.652)	0.653 (0.635-0.676)
**Striato-postcentral_left**	**0.662 (0.636-0.699)**	**0.693 (0.675-0.728)**
**Striato-postcentral_right**	**0.677 (0.650-0.704)**	**0.697 (0.678-0.722)**
**Striato-parietal_left**	**0.700 (0.675-0.721)**	**0.728 (0.719-0.753)**
**Striato-parietal_right**	**0.692 (0.677-0.713)**	**0.729 (0.710-0.740)**
Striato-occipital_left	0.701 (0.686-0.715)	0.715 (0.689-0.765)
**Striato-occipital_right**	**0.711 (0.691-0.732)**	**0.732 (0.700-0.761)**
**Global WM metric value**	**0.698 (0.681-0.711)**	**0.710 (0.700-0.728)**

I. DKI - MK

	SRC athletes, median (IQR) *n*=19	Controls, median (IQR) *n*=20
Arcuate_fascicle_left	0.929 (0.890-0.988)	0.992 (0.911-1.027)
Arcuate_fascicle_right	0.957 (0.912-1.010)	0.966 (0.941-1.028)
Anterior_Thalamic_Radiation_left	0.910 (0.827-0.937)	0.934 (0.884-0.983)
Anterior_Thalamic_Radiation_right	0.896 (0.849-0.933)	0.929 (0.891-0.963)
**Commissure_Anterior**	**0.745 (0.713-0.792)**	**0.799 (0.774-0.857)**
Rostrum	0.830 (0.772-0.870)	0.890 (0.809-0.929)
**Genu**	**0.902 (0.843-0.917)**	**0.926 (0.888-0.969)**
**Rostral_body_(Premotor)**	**0.998 (0.957-1.016)**	**1.040 (0.999-1.078)**
Anterior_midbody_(Primary_Motor)	1.048 (1.002-1.088)	1.078 (1.054-1.118)
Posterior_midbody_(Primary_Somatosensory)	0.998 (0.941-1.015)	1.010 (0.996-1.057)
Isthmus	0.912 (0.884-0.963)	0.931 (0.915-0.983)
Splenium	0.916 (0.891-0.973)	0.945 (0.911-0.976)
Cingulum_left	0.888 (0.826-0.911)	0.899 (0.884-0.961)
Cingulum_right	0.891 (0.839-0.918)	0.915 (0.875-0.964)
Corticospinal_tract_left	1.063 (1.006-1.111)	1.107 (1.075-1.147)
Corticospinal_tract_right	1.048 (0.995-1.093)	1.099 (1.056-1.131)
Middle_longitudinal_fascicle_left	0.897 (0.868-0.948)	0.930 (0.887-0.978)
Middle_longitudinal_fascicle_right	0.904 (0.884-0.964)	0.938 (0.889-0.965)
Fronto-pontine_tract_left	0.987 (0.925-1.035)	1.011 (0.986-1.067)
Fronto-pontine_tract_right	0.983 (0.901-1.014)	1.007 (0.975-1.063)
Fornix_left	0.631 (0.608-0.697)	0.667 (0.629-0.698)
Fornix_right	0.622 (0.597-0.681)	0.648 (0.612-0.695)
Inferior_cerebellar_peduncle_left	0.929 (0.850-0.986)	0.966 (0.876-1.027)
Inferior_cerebellar_peduncle_right	0.874 (0.805-1.011)	0.979 (0.917-1.014)
Inferior_occipito-frontal_fascicle_left	0.894 (0.850-0.927)	0.908 (0.882-0.961)
Inferior_occipito-frontal_fascicle_right	0.876 (0.859-0.915)	0.911 (0.883-0.953)
Inferior_longitudinal_fascicle_left	0.888 (0.825-0.946)	0.926 (0.877-0.971)
Inferior_longitudinal_fascicle_right	0.890 (0.848-0.955)	0.933 (0.883-0.967)
Middle_cerebellar_peduncle	0.933 (0.814-1.009)	0.976 (0.924-1.039)
Optic_radiation_left	0.948 (0.913-0.986)	0.961 (0.932-0.992)
Optic_radiation_right	0.927 (0.894-0.984)	0.970 (0.921-0.991)
Parieto_occipital_pontine_left	0.991 (0.953-1.024)	1.011 (0.995-1.061)
**Parieto_occipital_pontine_right**	**0.992 (0.963-1.014)**	**1.021 (0.997-1.051)**
**Superior_cerebellar_peduncle_left**	**0.930 (0.788-0.981)**	**0.964 (0.911-1.032)**
**Superior_cerebellar_peduncle_right**	**0.924 (0.806-0.954)**	**0.963 (0.919-0.987)**

Superior_longitudinal_fascicle_I_left	1.013 (0.967-1.066)	1.033 (1.006-1.094)
Superior_longitudinal_fascicle_I_right	1.016 (0.977-1.069)	1.046 (1.002-1.097)
Superior_longitudinal_fascicle_II_left	1.026 (0.990-1.065)	1.043 (1.025-1.104)
Superior_longitudinal_fascicle_II_right	1.000 (0.970-1.058)	1.030 (0.992-1.085)
Superior_longitudinal_fascicle_III_left	1.002 (0.976-1.067)	1.050 (0.990-1.086)
Superior_longitudinal_fascicle_III_right	0.987 (0.945-1.039)	1.007 (0.986-1.055)
Superior_Thalamic_Radiation_left	1.073 (0.966-1.110)	1.111 (1.073-1.136)
Superior_Thalamic_Radiation_right	1.078 (0.976-1.110)	1.115 (1.050-1.147)
Uncinate_fascicle_left	0.815 (0.777-0.832)	0.855 (0.813-0.905)
Uncinate_fascicle_right	0.810 (0.787-0.861)	0.843 (0.809-0.887)
Corpus_Callosum-all	0.911 (0.877-0.954)	0.940 (0.912-0.992)
Thalamo-prefrontal_left	0.944 (0.872-0.968)	0.979 (0.926-1.014)
Thalamo-prefrontal_right	0.935 (0.889-0.970)	0.965 (0.931-1.003)
Thalamo-premotor_left	1.009 (0.932-1.054)	1.043 (0.993-1.089)
Thalamo-premotor_right	1.004 (0.918-1.033)	1.036 (1.007-1.076)
Thalamo-precentral_left	1.065 (1.009-1.110)	1.098 (1.058-1.130)
Thalamo-precentral_right	1.033 (0.992-1.096)	1.086 (1.056-1.118)
Thalamo-postcentral_left	1.011 (0.965-1.050)	1.036 (1.005-1.080)
Thalamo-postcentral_right	1.011 (0.958-1.066)	1.047 (1.008-1.078)
Thalamo-parietal_left	0.952 (0.924-0.995)	0.974 (0.962-1.022)
Thalamo-parietal_right	0.949 (0.924-0.989)	0.984 (0.960-1.021)
Thalamo-occipital_left	0.941 (0.907-0.981)	0.955 (0.928-0.986)
Thalamo-occipital_right	0.918 (0.884-0.977)	0.961 (0.913-0.980)
Striato-fronto-orbital_left	0.874 (0.830-0.900)	0.893 (0.861-0.962)
Striato-fronto-orbital_right	0.855 (0.838-0.904)	0.916 (0.839-0.951)
Striato-prefrontal_left	0.918 (0.847-0.958)	0.948 (0.900-1.002)
Striato-prefrontal_right	0.921 (0.874-0.952)	0.948 (0.904-0.989)
Striato-premotor_left	1.018 (0.944-1.063)	1.043 (0.998-1.108)
Striato-premotor_right	1.023 (0.950-1.065)	1.051 (1.023-1.104)
Striato-precentral_left	1.045 (1.006.-1.099)	1.085 (1.036-1.117)
Striato-precentral_right	1.028 (0.974-1.093)	1.069 (1.046-1.111)
Striato-postcentral_left	0.995 (0.956-1.042)	1.031 (0.988-1.078)
Striato-postcentral_right	0.995 (0.952-1.061)	1.028 (1.001-1.073)
Striato-parietal_left	0.944 (0.922-0.993)	0.970 (0.952-1.019)
Striato-parietal_right	0.947 (0.929-0.997)	0.987 (0.962-1.019)
Striato-occipital_left	0.948 (0.902-9.987)	0.952 (0.934-0.997)
Striato-occipital_right	0.924 (0.885-0.994)	0.962 (0.920-0.990)
Global WM metric value	0.949 (0.898-0.976)	0.966 (0.945-1.017)

J. DKI - AK

	SRC athletes, median (IQR) *n*=19	Controls, median (IQR) *n*=20
**Arcuate_fascicle_left**	**0.899 (0.878-0.916)**	**0.863 (0.843-0.881)**
Arcuate_fascicle_right	0.887 (0.876-0.903)	0.873 (0.851-0.892)
**Anterior_Thalamic_Radiation_left**	**0.882 (0.868-0.903)**	**0.858 (0.831-0.875)**
Anterior_Thalamic_Radiation_right	0.868 (0.846-0.880)	0.848 (0.834-0.866)
Commissure_Anterior	0.754 (0.736-0.782)	0.743 (0.714-0.761)

Rostrum	0.779 (0.750-0.802)	0.759 (0.734-0.779)
**Genu**	**0.843 (0.829-0.859)**	**0.820 (0.790-0.835)**
**Rostral_body_(Premotor)**	**0.835 (0.819-0.855)**	**0.815 (0.799-0.834)**
**Anterior_midbody_(Primary_Motor)**	**0.873 (0.867-0.896)**	**0.861 (0.839-0.880)**
Posterior_midbody_(Primary_Somatosensory)	0.861 (0.853-0.873)	0.841 (0.833-0.870)
**Isthmus**	**0.841 (0.831-0.848)**	**0.814 (0.799-0.837)**
Splenium	0.829 (0.793-0.840)	0.811 (0.788-0.829)
**Cingulum_left**	**0.861 (0.845-0.884)**	**0.836 (0.812-0.862)**
**Cingulum_right**	**0.860 (0.847-0.881)**	**0.836 (0.811-0.856)**
**Corticospinal_tract_left**	**0.886 (0.871-0.896)**	**0.869 (0.859-0.879)**
Corticospinal_tract_right	0.875 (0.864-0.887)	0.869 (0.853-0.879)
**Middle_longitudinal_fascicle_left**	**0.871 (0.845-0.882)**	**0.829 (0.817-0.856)**
Middle_longitudinal_fascicle_right	0.858 (0.842-0.874)	0.845 (0.825-0.865)
**Fronto-pontine_tract_left**	**0.866 (0.852-0.881)**	**0.849 (0.839-0.864)**
Fronto-pontine_tract_right	0.857 (0.842-0.873)	0.851 (0.835-0.857)
Fornix_left	0.571 (0.555-0.615)	0.571 (0.561-0.593)
Fornix_right	0.571 (0.538-0.606)	0.563 (0.543-0.580)
Inferior_cerebellar_peduncle_left	0.966 (0.925-0.989)	0.955 (0.938-0.974)
Inferior_cerebellar_peduncle_right	0.948 (0.927-0.999)	0.969 (0.961-1.006)
**Inferior_occipito-frontal_fascicle_left**	**0.857 (0.842-0.866)**	**0.817 (0.804-0.843)**
**Inferior_occipito-frontal_fascicle_right**	**0.859 (0.844-0.870)**	**0.832 (0.814-0.858)**
**Inferior_longitudinal_fascicle_left**	**0.840 (0.800-0.855)**	**0.800 (0.787-0.826)**
Inferior_longitudinal_fascicle_right	0.852 (0.818-0.864)	0.825 (0.809-0.861)
Middle_cerebellar_peduncle	0.945 (0.925-0.967)	0.947 (0.918-0.963)
**Optic_radiation_left**	**0.848 (0.825-0.865)**	**0.823 (0.805-0.849)**
Optic_radiation_right	0.842 (0.834-0.865)	0.837 (0.812-0.857)
**Parieto_occipital_pontine_left**	**0.860 (0.848-0.865)**	**0.840 (0.827-0.851)**
Parieto_occipital_pontine_right	0.846 (0.839-0.852)	0.836 (0.819-0.850)
Superior_cerebellar_peduncle_left	0.866 (0.844-0.901)	0.871 (0.843-0.884)
Superior_cerebellar_peduncle_right	0.861 (0.843-0.901)	0.862 (0.849-0.912)
**Superior_longitudinal_fascicle_I_left**	**0.882 (0.866-0.894)**	**0.858 (0.832-0.886)**
Superior_longitudinal_fascicle_I_right	0.878 (0.859-0.885)	0.859 (0.838-0.886)
**Superior_longitudinal_fascicle_II_left**	**0.904 (0.890-0.920)**	**0.878 (0.858-0.900)**
Superior_longitudinal_fascicle_II_right	0.894 (0.880-0.910)	0.879 (0.856-0.900)
**Superior_longitudinal_fascicle_III_left**	**0.916 (0.898-0.945)**	**0.878 (0.859-0.906)**
Superior_longitudinal_fascicle_III_right	0.923 (0.881-0.934)	0.902 (0.879-0.923)
Superior_Thalamic_Radiation_left	0.885 (0.860-0.903)	0.873 (0.859-0.890)
Superior_Thalamic_Radiation_right	0.879 (0.865-0.895)	0.869 (0.855-0.881)
Uncinate_fascicle_left	0.780 (0.761-0.819)	0.759 (0.728-0.794)
Uncinate_fascicle_right	0.797 (0.782-0.820)	0.786 (0.757-0.803)
**Corpus_Callosum-all**	**0.858 (0.848-0.867)**	**0.834 (0.811-0.849)**
**Thalamo-prefrontal_left**	**0.884 (0.860-0.894)**	**0.853 (0.831-0.868)**
**Thalamo-prefrontal_right**	**0.869 (0.850-0.877)**	**0.846 (0.831-0.860)**
**Thalamo-premotor_left**	**0.897 (0.865-0.907)**	**0.863 (0.847-0.888)**
Thalamo-premotor_right	0.883 (0.876-0.903)	0.878 (0.859-0.888)
**Thalamo-precentral_left**	**0.909 (0.883-0.919)**	**0.882 (0.865-0.908)**
Thalamo-precentral_right	0.892 (0.885-0.910)	0.883 (0.866-0.901)

Thalamo-postcentral_left	0.884 (0.865-0.894)	0.867 (0.846-0.884)
Thalamo-postcentral_right	0.878 (0.864-0.893)	0.866 (0.848-0.875)
**Thalamo-parietal_left**	**0.867 (0.854-0.874)**	**0.842 (0.824-0.860)**
Thalamo-parietal_right	0.854 (0.846-0.860)	0.836 (0.818-0.859)
**Thalamo-occipital_left**	**0.848 (0.828-0.864)**	**0.823 (0.805-0.849)**
Thalamo-occipital_right	0.845 (0.838-0.869)	0.838 (0.817-0.860)
**Striato-fronto-orbital_left**	**0.848 (0.830-0.876)**	**0.827 (0.801-0.847)**
Striato-fronto-orbital_right	0.823 (0.804-0.850)	0.813 (0.790-0.831)
**Striato-prefrontal_left**	**0.884 (0.860-0.895)**	**0.851 (0.823-0.867)**
**Striato-prefrontal_right**	**0.868 (0.850-0.876)**	**0.846 (0.821-0.860)**
**Striato-premotor_left**	**0.906 (0.875-0.934)**	**0.871 (0.853-0.893)**
Striato-premotor_right	0.894 (0.869-0.921)	0.881 (0.859-0.894)
**Striato-precentral_left**	**0.908 (0.886-0.928)**	**0.887 (0.866-0.911)**
Striato-precentral_right	0.895 (0.885-0.922)	0.885 (0.866-0.908)
**Striato-postcentral_left**	**0.893 (0.882-0.906)**	**0.872 (0.854-0.888)**
**Striato-postcentral_right**	**0.885 (0.875-0.907)**	**0.873 (0.851-0.893)**
**Striato-parietal_left**	**0.862 (0.851-0.875)**	**0.836 (0.816-0.859)**
Striato-parietal_right	0.860 (0.849-0.875)	0.841 (0.823-0.869)
**Striato-occipital_left**	**0.834 (0.827-0.851)**	**0.807 (0.793-0.833)**
Striato-occipital_right	0.852 (0.833-0.868)	0.831 (0.811-0.855)
**Global WM metric value**	**0.861 (0.851-0.872)**	**0.842 (0.827-0.860)**

**K. DKI - RK**		
	SRC athletes, median (IQR) *n*=19	Controls, median (IQR) *n*=20
Arcuate_fascicle_left	1.080 (0.958-1.166)	1.199 (1.087-1.269)
Arcuate_fascicle_right	1.066 (1.051-1.186)	1.144 (1.086-1.251)
**Anterior_Thalamic_Radiation_left**	**0.995 (0.829-1.033)**	**1.073 (1.007-1.176)**
**Anterior_Thalamic_Radiation_right**	**0.977 (0.895-1.019)**	**1.058 (0.994-1.120)**
**Commissure_Anterior**	**0.970 (0.725-0.883)**	**0.890 (0.829-0.992)**
Rostrum	0.911 (0.803-1.019)	1.045 (0.919-1.170)
**Genu**	**1.036 (0.923-1.110)**	**1.114 (1.096-1.200)**
**Rostral_body_(Premotor)**	**1.229 (1.150-1.331)**	**1.358 (1.301-1.432)**
Anterior_midbody_(Primary_Motor)	1.394 (1.229-1.459)	1.455 (1.375-1.550)
Posterior_midbody_(Primary_Somatosensory)	1.291 (1.136-1.355)	1.348 (1.276-1.469)
Isthmus	1.080 (1.019-1.160)	1.146 (1.098-1.238)
Splenium	1.101 (1.016-1.188)	1.152 (1.107-1.209)
Cingulum_left	1.026 (0.868-1.080)	1.110 (1.037-1.192)
**Cingulum_right**	**1.016 (0.883-1.081)**	**1.096 (1.034-1.166)**
Corticospinal_tract_left	1.310 (1.185-1.475)	1.444 (1.382-1.548)
Corticospinal_tract_right	1.325 (1.206-1.461)	1.445 (1.370-1.515)
Middle_longitudinal_fascicle_left	1.012 (0.979-1.134)	1.136 (1.033-1.212)
Middle_longitudinal_fascicle_right	1.081 (0.980-1.116)	1.097 (1.049-1.170)
Fronto-pontine_tract_left	1.182 (1.044-1.278)	1.273 (1.228-1.361)
Fronto-pontine_tract_right	1.154 (1.010-1.276)	1.260 (1.197-1.361)
Fornix_left	0.660 (0.626-0.825)	0.747 (0.683-0.841)
Fornix_right	0.631 (0.591-0.779)	0.749 (0.666-0.828)

**Inferior_cerebellar_peduncle_left**	**0.947 (0.755-1.045)**	**1.099 (0.864.1.150)**
Inferior_cerebellar_peduncle_right	0.946 (0.752-1.066)	1.048 (0.925-1.167)
**Inferior_occipito-frontal_fascicle_left**	**0.998 (0.931-1.089)**	**1.079 (1.039-1.159)**
**Inferior_occipito-frontal_fascicle_right**	**0.964 (0.911-1.049)**	**1.045 (1.007-1.118)**
**Inferior_longitudinal_fascicle_left**	**1.044 (0.878-1.177)**	**1.142 (1.065-1.218)**
**Inferior_longitudinal_fascicle_right**	**1.012 (0.902-1.127)**	**1.077 (1.033-1.176)**
**Middle_cerebellar_peduncle**	**0.978 (0.828-1.183)**	**1.089 (0.989-1.223)**
Optic_radiation_left	1.120 (1.041-1.191)	1.189 (1.148-1.236)
**Optic_radiation_right**	**1.096 (1.007-1.176)**	**1.163 (1.115-1.232)**
Parieto_occipital_pontine_left	1.218 (1.116-1.295)	1.287 (1.210-1.367)
**Parieto_occipital_pontine_right**	**1.253 (1.158-1.295)**	**1.324 (1.260-1.392)**
**Superior_cerebellar_peduncle_left**	**0.935 (0.699-1.089)**	**1.060 (0.973-1.164)**
**Superior_cerebellar_peduncle_right**	**0.945 (0.746-1.068)**	**1.093 (0.973-1.135)**
Superior_longitudinal_fascicle_I_left	1.298 (1.108-1.383)	1.383 (1.264-1.439)
Superior_longitudinal_fascicle_I_right	1.331 (1.120-1.385)	1.359 (1.264-1.437)
Superior_longitudinal_fascicle_II_left	1.281 (1.144-1.345)	1.322 (1.243-1.430)
Superior_longitudinal_fascicle_II_right	1.232 (1.124-1.278)	1.287 (1.216-1.361)
Superior_longitudinal_fascicle_III_left	1.201 (1.090-1.299)	1.286 (1.201-1.342)
Superior_longitudinal_fascicle_III_right	1.160 (1.093-1.227)	1.210 (1.161-1.257)
Superior_Thalamic_Radiation_left	1.335 (1.065-1.500)	1.458 (1.349-1.547)
Superior_Thalamic_Radiation_right	1.385 (1.148-1.539)	1.491 (1.357-1.586)
**Uncinate_fascicle_left**	**0.914 (0.791-0.979)**	**1.005 (0.910-1.117)**
Uncinate_fascicle_right	0.902 (0.871-0.948)	0.975 (0.891-1.083)
**Corpus_Callosum-all**	**1.071 (0.927-1.153)**	**1.137 (1.105-1.218)**
Thalamo-prefrontal_left	1.071 (0.927-1.153)	1.171 (1.103-1.258)
**Thalamo-prefrontal_right**	**1.052 (0.954-1.128)**	**1.138 (1.076-1.212)**
**Thalamo-premotor_left**	**1.208 (0.998-1.296)**	**1.281 (1.188-1.358)**
**Thalamo-premotor_right**	**1.171 (1.045-1.264)**	**1.280 (1.226-1.324)**
Thalamo-precentral_left	1.354 (1.152-1.454)	1.433 (1.336-1.508)
Thalamo-precentral_right	1.337 (1.161-1.429)	1.414 (1.328-1.455)
Thalamo-postcentral_left	1.257 (1.069-1.348)	1.312 (1.219-1.393)
Thalamo-postcentral_right	1.264 (1.103-1.357)	1.339 (1.264-1.409)
Thalamo-parietal_left	1.159 (1.064-1.233)	1.211 (1.152-1.295)
**Thalamo-parietal_right**	**1.141 (1.100-1.243)**	**1.240 (1.170-1.314)**
Thalamo-occipital_left	1.104 (1.025-1.178)	1.175 (1.134-1.225)
**Thalamo-occipital_right**	**1.087 (0.987-1.156)**	**1.134 (1.098-1.211)**
**Striato-fronto-orbital_left**	**0.983 (0.871-1.024)**	**1.034 (0.988-1.171)**
Striato-fronto-orbital_right	0.966 (0.883-1.046)	1.061 (0.918-1.161)
**Striato-prefrontal_left**	**1.050 (0.888-1.110)**	**1.136 (1.068-1.233)**
**Striato-prefrontal_right**	**1.019 (0.947-1.128)**	**1.126 (1.074-1.214)**
**Striato-premotor_left**	**1.238 (1.051-1.340)**	**1.310 (1.258-1.432)**
**Striato-premotor_right**	**1.225 (1.122-1.330)**	**1.327 (1.270-1.396)**
Striato-precentral_left	1.367 (1.160-1.415)	1.414 (1.327-1.491)
Striato-precentral_right	1.339 (1.131-1.417)	1.392 (1.337-1.458)
Striato-postcentral_left	1.267 (1.110-1.336)	1.326 (1.204-1.389)
Striato-postcentral_right	1.282 (1.075-1.344)	1.320 (1.272-1.390)
Striato-parietal_left	1.155 (1.060-1.228)	1.221 (1.153-1.299)

Striato-parietal_right	1.145 (1.106-1.244)	1.232 (1.192-1.319)
Striato-occipital_left	1.172 (1.052-1.226)	1.202 (1.160-1.256)
**Striato-occipital_right**	**1.117 (1.000-1.181)**	**1.147 (1.116-1.227)**
**Global WM metric value**	**1.121 (1.008-1.182)**	**1.185 (1.134-1.263)**

Supplementary Table 2: DTI and DKI metrics

The median and interquartile range (IQR) for each diffusion metric, in the SRC athletes and controls, in the 72 anatomic structures that were available with TractSeg analysis and one global white matter (WM) metric value for all the tracts are presented in Table A to K. Table A to D contains the diffusion tensor imaging (DTI) metrics fractional anisotropy (FA), mean diffusivity (MD), axial diffusivity (AD) and radial diffusivity (RD), and table E to K the diffusion kurtosis imaging (DKI) metrics FA, MD, AD, RD, mean kurtosis (MK), axial kurtosis (AK) and radial kurtosis (RK). The metrics are compared between groups with Mann-Whitney U test and P-values are considered significant and highlighted in bold type. If SRC athletes has a higher metric compared with the controls it is marked with "+", and if they had a lower metric it is marked with "-".
